# Search for single production of vector-like quarks decaying to a top quark and a $\mathrm {W}$ boson in proton–proton collisions at $\sqrt{s} = 13 \,\text {TeV}$

**DOI:** 10.1140/epjc/s10052-019-6556-3

**Published:** 2019-01-30

**Authors:** A. M. Sirunyan, A. Tumasyan, W. Adam, F. Ambrogi, E. Asilar, T. Bergauer, J. Brandstetter, M. Dragicevic, J. Erö, A. Escalante Del Valle, M. Flechl, R. Frühwirth, V. M. Ghete, J. Hrubec, M. Jeitler, N. Krammer, I. Krätschmer, D. Liko, T. Madlener, I. Mikulec, N. Rad, H. Rohringer, J. Schieck, R. Schöfbeck, M. Spanring, D. Spitzbart, A. Taurok, W. Waltenberger, J. Wittmann, C.-E. Wulz, M. Zarucki, V. Chekhovsky, V. Mossolov, J. Suarez Gonzalez, E. A. De Wolf, D. Di Croce, X. Janssen, J. Lauwers, M. Pieters, H. Van Haevermaet, P. Van Mechelen, N. Van Remortel, S. Abu Zeid, F. Blekman, J. D’Hondt, J. De Clercq, K. Deroover, G. Flouris, D. Lontkovskyi, S. Lowette, I. Marchesini, S. Moortgat, L. Moreels, Q. Python, K. Skovpen, S. Tavernier, W. Van Doninck, P. Van Mulders, I. Van Parijs, D. Beghin, B. Bilin, H. Brun, B. Clerbaux, G. De Lentdecker, H. Delannoy, B. Dorney, G. Fasanella, L. Favart, R. Goldouzian, A. Grebenyuk, A. K. Kalsi, T. Lenzi, J. Luetic, N. Postiau, E. Starling, L. Thomas, C. Vander Velde, P. Vanlaer, D. Vannerom, Q. Wang, T. Cornelis, D. Dobur, A. Fagot, M. Gul, I. Khvastunov, D. Poyraz, C. Roskas, D. Trocino, M. Tytgat, W. Verbeke, B. Vermassen, M. Vit, N. Zaganidis, H. Bakhshiansohi, O. Bondu, S. Brochet, G. Bruno, C. Caputo, P. David, C. Delaere, M. Delcourt, A. Giammanco, G. Krintiras, V. Lemaitre, A. Magitteri, A. Mertens, K. Piotrzkowski, A. Saggio, M. Vidal Marono, S. Wertz, J. Zobec, F. L. Alves, G. A. Alves, M Correa Martins Junior, G. Correia Silva, C. Hensel, A. Moraes, M. E. Pol, P. Rebello Teles, E. Belchior Batista Das Chagas, W. Carvalho, J. Chinellato, E. Coelho, E. M. Da Costa, G. G. Da Silveira, D. De Jesus Damiao, C. De Oliveira Martins, S. Fonseca De Souza, H. Malbouisson, D. Matos Figueiredo, M. Melo De Almeida, C. Mora Herrera, L. Mundim, H. Nogima, W. L. Prado Da Silva, L. J. Sanchez Rosas, A. Santoro, A. Sznajder, M. Thiel, E. J. Tonelli Manganote, F. Torres Da Silva De Araujo, A. Vilela Pereira, S. Ahuja, C. A. Bernardes, L. Calligaris, T. R. Fernandez Perez Tomei, E. M. Gregores, P. G. Mercadante, S. F. Novaes, Sandra S. Padula, A. Aleksandrov, R. Hadjiiska, P. Iaydjiev, A. Marinov, M. Misheva, M. Rodozov, M. Shopova, G. Sultanov, A. Dimitrov, L. Litov, B. Pavlov, P. Petkov, W. Fang, X. Gao, L. Yuan, M. Ahmad, J. G. Bian, G. M. Chen, H. S. Chen, M. Chen, Y. Chen, C. H. Jiang, D. Leggat, H. Liao, Z. Liu, F. Romeo, S. M. Shaheen, A. Spiezia, J. Tao, Z. Wang, E. Yazgan, H. Zhang, S. Zhang, J. Zhao, Y. Ban, G. Chen, A. Levin, J. Li, L. Li, Q. Li, Y. Mao, S. J. Qian, D. Wang, Y. Wang, C. Avila, A. Cabrera, C. A. Carrillo Montoya, L. F. Chaparro Sierra, C. Florez, C. F. González Hernández, M. A. Segura Delgado, B. Courbon, N. Godinovic, D. Lelas, I. Puljak, T. Sculac, Z. Antunovic, M. Kovac, V. Brigljevic, D. Ferencek, K. Kadija, B. Mesic, A. Starodumov, T. Susa, M. W. Ather, A. Attikis, M. Kolosova, G. Mavromanolakis, J. Mousa, C. Nicolaou, F. Ptochos, P. A. Razis, H. Rykaczewski, M. Finger, M. Finger, E. Ayala, E. Carrera Jarrin, M. A. Mahmoud, A. Mahrous, Y. Mohammed, S. Bhowmik, A. Carvalho Antunes De Oliveira, R. K. Dewanjee, K. Ehataht, M. Kadastik, M. Raidal, C. Veelken, P. Eerola, H. Kirschenmann, J. Pekkanen, M. Voutilainen, J. Havukainen, J. K. Heikkilä, T. Järvinen, V. Karimäki, R. Kinnunen, T. Lampén, K. Lassila-Perini, S. Laurila, S. Lehti, T. Lindén, P. Luukka, T. Mäenpää, H. Siikonen, E. Tuominen, J. Tuominiemi, T. Tuuva, M. Besancon, F. Couderc, M. Dejardin, D. Denegri, J. L. Faure, F. Ferri, S. Ganjour, A. Givernaud, P. Gras, G. Hamel de Monchenault, P. Jarry, C. Leloup, E. Locci, J. Malcles, G. Negro, J. Rander, A. Rosowsky, M. Ö. Sahin, M. Titov, A. Abdulsalam, C. Amendola, I. Antropov, F. Beaudette, P. Busson, C. Charlot, R. Granier de Cassagnac, I. Kucher, A. Lobanov, J. Martin Blanco, C. Martin Perez, M. Nguyen, C. Ochando, G. Ortona, P. Paganini, P. Pigard, J. Rembser, R. Salerno, J. B. Sauvan, Y. Sirois, A. G. Stahl Leiton, A. Zabi, A. Zghiche, J.-L. Agram, J. Andrea, D. Bloch, J.-M. Brom, E. C. Chabert, V Cherepanov, C. Collard, E. Conte, J.-C. Fontaine, D. Gelé, U. Goerlach, M. Jansová, A.-C. Le Bihan, N. Tonon, P. Van Hove, S. Gadrat, S. Beauceron, C. Bernet, G. Boudoul, N. Chanon, R. Chierici, D. Contardo, P. Depasse, H. El Mamouni, J. Fay, L. Finco, S. Gascon, M. Gouzevitch, G. Grenier, B. Ille, F. Lagarde, I. B. Laktineh, H. Lattaud, M. Lethuillier, L. Mirabito, S. Perries, A. Popov, V. Sordini, G. Touquet, M. Vander Donckt, S. Viret, A. Khvedelidze, Z. Tsamalaidze, C. Autermann, L. Feld, M. K. Kiesel, K. Klein, M. Lipinski, M. Preuten, M. P. Rauch, C. Schomakers, J. Schulz, M. Teroerde, B. Wittmer, A. Albert, D. Duchardt, M. Erdmann, S. Erdweg, T. Esch, R. Fischer, S. Ghosh, A. Güth, T. Hebbeker, C. Heidemann, K. Hoepfner, H. Keller, L. Mastrolorenzo, M. Merschmeyer, A. Meyer, P. Millet, S. Mukherjee, T. Pook, M. Radziej, H. Reithler, M. Rieger, A. Schmidt, D. Teyssier, S. Thüer, G. Flügge, O. Hlushchenko, T. Kress, A. Künsken, T. Müller, A. Nehrkorn, A. Nowack, C. Pistone, O. Pooth, D. Roy, H. Sert, A. Stahl, M. Aldaya Martin, T. Arndt, C. Asawatangtrakuldee, I. Babounikau, K. Beernaert, O. Behnke, U. Behrens, A. Bermúdez Martínez, D. Bertsche, A. A. Bin Anuar, K. Borras, V. Botta, A. Campbell, P. Connor, C. Contreras-Campana, V. Danilov, A. De Wit, M. M. Defranchis, C. Diez Pardos, D. Domínguez Damiani, G. Eckerlin, T. Eichhorn, A. Elwood, E. Eren, E. Gallo, A. Geiser, J. M. Grados Luyando, A. Grohsjean, M. Guthoff, M. Haranko, A. Harb, J. Hauk, H. Jung, M. Kasemann, J. Keaveney, C. Kleinwort, J. Knolle, D. Krücker, W. Lange, A. Lelek, T. Lenz, J. Leonard, K. Lipka, W. Lohmann, R. Mankel, I.-A. Melzer-Pellmann, A. B. Meyer, M. Meyer, M. Missiroli, G. Mittag, J. Mnich, V. Myronenko, S. K. Pflitsch, D. Pitzl, A. Raspereza, M. Savitskyi, P. Saxena, P. Schütze, C. Schwanenberger, R. Shevchenko, A. Singh, H. Tholen, O. Turkot, A. Vagnerini, G. P. Van Onsem, R. Walsh, Y. Wen, K. Wichmann, C. Wissing, O. Zenaiev, R. Aggleton, S. Bein, L. Benato, A. Benecke, V. Blobel, T. Dreyer, A. Ebrahimi, E. Garutti, D. Gonzalez, P. Gunnellini, J. Haller, A. Hinzmann, A. Karavdina, G. Kasieczka, R. Klanner, R. Kogler, N. Kovalchuk, S. Kurz, V. Kutzner, J. Lange, D. Marconi, J. Multhaup, M. Niedziela, C. E. N. Niemeyer, D. Nowatschin, A. Perieanu, A. Reimers, O. Rieger, C. Scharf, P. Schleper, S. Schumann, J. Schwandt, J. Sonneveld, H. Stadie, G. Steinbrück, F. M. Stober, M. Stöver, A. Vanhoefer, B. Vormwald, I. Zoi, M. Akbiyik, C. Barth, M. Baselga, S. Baur, E. Butz, R. Caspart, T. Chwalek, F. Colombo, W. De Boer, A. Dierlamm, K. El Morabit, N. Faltermann, B. Freund, M. Giffels, M. A. Harrendorf, F. Hartmann, S. M. Heindl, U. Husemann, I. Katkov, S. Kudella, S. Mitra, M. U. Mozer, Th. Müller, M. Musich, M. Plagge, G. Quast, K. Rabbertz, M. Schröder, I. Shvetsov, H. J. Simonis, R. Ulrich, S. Wayand, M. Weber, T. Weiler, C. Wöhrmann, R. Wolf, G. Anagnostou, G. Daskalakis, T. Geralis, A. Kyriakis, D. Loukas, G. Paspalaki, I. Topsis-Giotis, G. Karathanasis, S. Kesisoglou, P. Kontaxakis, A. Panagiotou, I. Papavergou, N. Saoulidou, E. Tziaferi, K. Vellidis, K. Kousouris, I. Papakrivopoulos, G. Tsipolitis, I. Evangelou, C. Foudas, P. Gianneios, P. Katsoulis, P. Kokkas, S. Mallios, N. Manthos, I. Papadopoulos, E. Paradas, J. Strologas, F. A. Triantis, D. Tsitsonis, M. Bartók, M. Csanad, N. Filipovic, P. Major, M. I. Nagy, G. Pasztor, O. Surányi, G. I. Veres, G. Bencze, C. Hajdu, D. Horvath, Á. Hunyadi, F. Sikler, T. Á. Vámi, V. Veszpremi, G. Vesztergombi, N. Beni, S. Czellar, J. Karancsi, A. Makovec, J. Molnar, Z. Szillasi, P. Raics, Z. L. Trocsanyi, B. Ujvari, S. Choudhury, J. R. Komaragiri, P. C. Tiwari, S. Bahinipati, C. Kar, P. Mal, K. Mandal, A. Nayak, D. K. Sahoo, S. K. Swain, S. Bansal, S. B. Beri, V. Bhatnagar, S. Chauhan, R. Chawla, N. Dhingra, R. Gupta, A. Kaur, M. Kaur, S. Kaur, P. Kumari, M. Lohan, A. Mehta, K. Sandeep, S. Sharma, J. B. Singh, A. K. Virdi, G. Walia, A. Bhardwaj, B. C. Choudhary, R. B. Garg, M. Gola, S. Keshri, Ashok Kumar, S. Malhotra, M. Naimuddin, P. Priyanka, K. Ranjan, Aashaq Shah, R. Sharma, R. Bhardwaj, M. Bharti, R. Bhattacharya, S. Bhattacharya, U. Bhawandeep, D. Bhowmik, S. Dey, S. Dutt, S. Dutta, S. Ghosh, K. Mondal, S. Nandan, A. Purohit, P. K. Rout, A. Roy, S. Roy Chowdhury, G. Saha, S. Sarkar, M. Sharan, B. Singh, S. Thakur, P. K. Behera, R. Chudasama, D. Dutta, V. Jha, V. Kumar, P. K. Netrakanti, L. M. Pant, P. Shukla, T. Aziz, M. A. Bhat, S. Dugad, G. B. Mohanty, N. Sur, B. Sutar, RavindraKumar Verma, S. Banerjee, S. Bhattacharya, S. Chatterjee, P. Das, M. Guchait, Sa. Jain, S. Karmakar, S. Kumar, M. Maity, G. Majumder, K. Mazumdar, N. Sahoo, T. Sarkar, S. Chauhan, S. Dube, V. Hegde, A. Kapoor, K. Kothekar, S. Pandey, A. Rane, S. Sharma, S. Chenarani, E. Eskandari Tadavani, S. M. Etesami, M. Khakzad, M. Mohammadi Najafabadi, M. Naseri, F. Rezaei Hosseinabadi, B. Safarzadeh, M. Zeinali, M. Felcini, M. Grunewald, M. Abbrescia, C. Calabria, A. Colaleo, D. Creanza, L. Cristella, N. De Filippis, M. De Palma, A. Di Florio, F. Errico, L. Fiore, A. Gelmi, G. Iaselli, M. Ince, S. Lezki, G. Maggi, M. Maggi, G. Miniello, S. My, S. Nuzzo, A. Pompili, G. Pugliese, R. Radogna, A. Ranieri, G. Selvaggi, A. Sharma, L. Silvestris, R. Venditti, P. Verwilligen, G. Zito, G. Abbiendi, C. Battilana, D. Bonacorsi, L. Borgonovi, S. Braibant-Giacomelli, R. Campanini, P. Capiluppi, A. Castro, F. R. Cavallo, S. S. Chhibra, C. Ciocca, G. Codispoti, M. Cuffiani, G. M. Dallavalle, F. Fabbri, A. Fanfani, E. Fontanesi, P. Giacomelli, C. Grandi, L. Guiducci, S. Lo Meo, S. Marcellini, G. Masetti, A. Montanari, F. L. Navarria, A. Perrotta, F. Primavera, A. M. Rossi, T. Rovelli, G. P. Siroli, N. Tosi, S. Albergo, A. Di Mattia, R. Potenza, A. Tricomi, C. Tuve, G. Barbagli, K. Chatterjee, V. Ciulli, C. Civinini, R. D’Alessandro, E. Focardi, G. Latino, P. Lenzi, M. Meschini, S. Paoletti, L. Russo, G. Sguazzoni, D. Strom, L. Viliani, L. Benussi, S. Bianco, F. Fabbri, D. Piccolo, F. Ferro, R. Mulargia, F. Ravera, E. Robutti, S. Tosi, A. Benaglia, A. Beschi, F. Brivio, V. Ciriolo, S. Di Guida, M. E. Dinardo, S. Fiorendi, S. Gennai, A. Ghezzi, P. Govoni, M. Malberti, S. Malvezzi, A. Massironi, D. Menasce, F. Monti, L. Moroni, M. Paganoni, D. Pedrini, S. Ragazzi, T. Tabarelli de Fatis, D. Zuolo, S. Buontempo, N. Cavallo, A. De Iorio, A. Di Crescenzo, F. Fabozzi, F. Fienga, G. Galati, A. O. M. Iorio, W. A. Khan, L. Lista, S. Meola, P. Paolucci, C. Sciacca, E. Voevodina, P. Azzi, N. Bacchetta, A. Boletti, A. Bragagnolo, R. Carlin, P. Checchia, M. Dall’Osso, P. De Castro Manzano, T. Dorigo, U. Dosselli, F. Gasparini, U. Gasparini, A. Gozzelino, S. Y. Hoh, S. Lacaprara, P. Lujan, M. Margoni, A. T. Meneguzzo, J. Pazzini, N. Pozzobon, P. Ronchese, R. Rossin, F. Simonetto, A. Tiko, E. Torassa, M. Tosi, S. Ventura, M. Zanetti, P. Zotto, A. Braghieri, A. Magnani, P. Montagna, S. P. Ratti, V. Re, M. Ressegotti, C. Riccardi, P. Salvini, I. Vai, P. Vitulo, M. Biasini, G. M. Bilei, C. Cecchi, D. Ciangottini, L. Fanò, P. Lariccia, R. Leonardi, E. Manoni, G. Mantovani, V. Mariani, M. Menichelli, A. Rossi, A. Santocchia, D. Spiga, K. Androsov, P. Azzurri, G. Bagliesi, L. Bianchini, T. Boccali, L. Borrello, R. Castaldi, M. A. Ciocci, R. Dell’Orso, G. Fedi, F. Fiori, L. Giannini, A. Giassi, M. T. Grippo, F. Ligabue, E. Manca, G. Mandorli, A. Messineo, F. Palla, A. Rizzi, G. Rolandi, P. Spagnolo, R. Tenchini, G. Tonelli, A. Venturi, P. G. Verdini, L. Barone, F. Cavallari, M. Cipriani, D. Del Re, E. Di Marco, M. Diemoz, S. Gelli, E. Longo, B. Marzocchi, P. Meridiani, G. Organtini, F. Pandolfi, R. Paramatti, F. Preiato, S. Rahatlou, C. Rovelli, F. Santanastasio, N. Amapane, R. Arcidiacono, S. Argiro, M. Arneodo, N. Bartosik, R. Bellan, C. Biino, N. Cartiglia, F. Cenna, S. Cometti, M. Costa, R. Covarelli, N. Demaria, B. Kiani, C. Mariotti, S. Maselli, E. Migliore, V. Monaco, E. Monteil, M. Monteno, M. M. Obertino, L. Pacher, N. Pastrone, M. Pelliccioni, G. L. Pinna Angioni, A. Romero, M. Ruspa, R. Sacchi, K. Shchelina, V. Sola, A. Solano, D. Soldi, A. Staiano, S. Belforte, V. Candelise, M. Casarsa, F. Cossutti, A. Da Rold, G. Della Ricca, F. Vazzoler, A. Zanetti, D. H. Kim, G. N. Kim, M. S. Kim, J. Lee, S. Lee, S. W. Lee, C. S. Moon, Y. D. Oh, S. I. Pak, S. Sekmen, D. C. Son, Y. C. Yang, H. Kim, D. H. Moon, G. Oh, B. Francois, J. Goh, T. J. Kim, S. Cho, S. Choi, Y. Go, D. Gyun, S. Ha, B. Hong, Y. Jo, K. Lee, K. S. Lee, S. Lee, J. Lim, S. K. Park, Y. Roh, H. S. Kim, J. Almond, J. Kim, J. S. Kim, H. Lee, K. Lee, K. Nam, S. B. Oh, B. C. Radburn-Smith, S. h. Seo, U. K. Yang, H. D. Yoo, G. B. Yu, D. Jeon, H. Kim, J. H. Kim, J. S. H. Lee, I. C. Park, Y. Choi, C. Hwang, J. Lee, I. Yu, V. Dudenas, A. Juodagalvis, J. Vaitkus, I. Ahmed, Z. A. Ibrahim, M. A. B. Md Ali, F. Mohamad Idris, W. A. T. Wan Abdullah, M. N. Yusli, Z. Zolkapli, J. F. Benitez, A. Castaneda Hernandez, J. A. Murillo Quijada, H. Castilla-Valdez, E. De La Cruz-Burelo, M. C. Duran-Osuna, I. Heredia-De La Cruz, R. Lopez-Fernandez, J. Mejia Guisao, R. I. Rabadan-Trejo, M. Ramirez-Garcia, G. Ramirez-Sanchez, R. Reyes-Almanza, A. Sanchez-Hernandez, S. Carrillo Moreno, C. Oropeza Barrera, F. Vazquez Valencia, J. Eysermans, I. Pedraza, H. A. Salazar Ibarguen, C. Uribe Estrada, A. Morelos Pineda, D. Krofcheck, S. Bheesette, P. H. Butler, A. Ahmad, M. Ahmad, M. I. Asghar, Q. Hassan, H. R. Hoorani, A. Saddique, M. A. Shah, M. Shoaib, M. Waqas, H. Bialkowska, M. Bluj, B. Boimska, T. Frueboes, M. Górski, M. Kazana, M. Szleper, P. Traczyk, P. Zalewski, K. Bunkowski, A. Byszuk, K. Doroba, A. Kalinowski, M. Konecki, J. Krolikowski, M. Misiura, M. Olszewski, A. Pyskir, M. Walczak, M. Araujo, P. Bargassa, C. Beirão Da Cruz E Silva, A. Di Francesco, P. Faccioli, B. Galinhas, M. Gallinaro, J. Hollar, N. Leonardo, J. Seixas, G. Strong, O. Toldaiev, J. Varela, S. Afanasiev, P. Bunin, M. Gavrilenko, I. Golutvin, I. Gorbunov, A. Kamenev, V. Karjavine, A. Lanev, A. Malakhov, V. Matveev, P. Moisenz, V. Palichik, V. Perelygin, S. Shmatov, S. Shulha, N. Skatchkov, V. Smirnov, N. Voytishin, A. Zarubin, V. Golovtsov, Y. Ivanov, V. Kim, E. Kuznetsova, P. Levchenko, V. Murzin, V. Oreshkin, I. Smirnov, D. Sosnov, V. Sulimov, L. Uvarov, S. Vavilov, A. Vorobyev, Yu. Andreev, A. Dermenev, S. Gninenko, N. Golubev, A. Karneyeu, M. Kirsanov, N. Krasnikov, A. Pashenkov, D. Tlisov, A. Toropin, V. Epshteyn, V. Gavrilov, N. Lychkovskaya, V. Popov, I. Pozdnyakov, G. Safronov, A. Spiridonov, A. Stepennov, V. Stolin, M. Toms, E. Vlasov, A. Zhokin, T. Aushev, M. Chadeeva, P. Parygin, D. Philippov, S. Polikarpov, E. Popova, V. Rusinov, V. Andreev, M. Azarkin, I. Dremin, M. Kirakosyan, A. Terkulov, A. Baskakov, A. Belyaev, E. Boos, V. Bunichev, M. Dubinin, L. Dudko, A. Ershov, V. Klyukhin, N. Korneeva, I. Lokhtin, I. Miagkov, S. Obraztsov, M. Perfilov, V. Savrin, P. Volkov, A. Barnyakov, V. Blinov, T. Dimova, L. Kardapoltsev, Y. Skovpen, I. Azhgirey, I. Bayshev, S. Bitioukov, D. Elumakhov, A. Godizov, V. Kachanov, A. Kalinin, D. Konstantinov, P. Mandrik, V. Petrov, R. Ryutin, S. Slabospitskii, A. Sobol, S. Troshin, N. Tyurin, A. Uzunian, A. Volkov, A. Babaev, S. Baidali, V. Okhotnikov, P. Adzic, P. Cirkovic, D. Devetak, M. Dordevic, J. Milosevic, J. Alcaraz Maestre, A. Álvarez Fernández, I. Bachiller, M. Barrio Luna, J. A. Brochero Cifuentes, M. Cerrada, N. Colino, B. De La Cruz, A. Delgado Peris, C. Fernandez Bedoya, J. P. Fernández Ramos, J. Flix, M. C. Fouz, O. Gonzalez Lopez, S. Goy Lopez, J. M. Hernandez, M. I. Josa, D. Moran, A. Pérez-Calero Yzquierdo, J. Puerta Pelayo, I. Redondo, L. Romero, M. S. Soares, A. Triossi, C. Albajar, J. F. de Trocóniz, J. Cuevas, C. Erice, J. Fernandez Menendez, S. Folgueras, I. Gonzalez Caballero, J. R. González Fernández, E. Palencia Cortezon, V. Rodríguez Bouza, S. Sanchez Cruz, P. Vischia, J. M. Vizan Garcia, I. J. Cabrillo, A. Calderon, B. Chazin Quero, J. Duarte Campderros, M. Fernandez, P. J. Fernández Manteca, A. García Alonso, J. Garcia-Ferrero, G. Gomez, A. Lopez Virto, J. Marco, C. Martinez Rivero, P. Martinez Ruiz del Arbol, F. Matorras, J. Piedra Gomez, C. Prieels, T. Rodrigo, A. Ruiz-Jimeno, L. Scodellaro, N. Trevisani, I. Vila, R. Vilar Cortabitarte, N. Wickramage, D. Abbaneo, B. Akgun, E. Auffray, G. Auzinger, P. Baillon, A. H. Ball, D. Barney, J. Bendavid, M. Bianco, A. Bocci, C. Botta, E. Brondolin, T. Camporesi, M. Cepeda, G. Cerminara, E. Chapon, Y. Chen, G. Cucciati, D. d’Enterria, A. Dabrowski, N. Daci, V. Daponte, A. David, A. De Roeck, N. Deelen, M. Dobson, M. Dünser, N. Dupont, A. Elliott-Peisert, P. Everaerts, F. Fallavollita, D. Fasanella, G. Franzoni, J. Fulcher, W. Funk, D. Gigi, A. Gilbert, K. Gill, F. Glege, M. Gruchala, M. Guilbaud, D. Gulhan, J. Hegeman, C. Heidegger, V. Innocente, A. Jafari, P. Janot, O. Karacheban, J. Kieseler, A. Kornmayer, M. Krammer, C. Lange, P. Lecoq, C. Lourenço, L. Malgeri, M. Mannelli, F. Meijers, J. A. Merlin, S. Mersi, E. Meschi, P. Milenovic, F. Moortgat, M. Mulders, J. Ngadiuba, S. Nourbakhsh, S. Orfanelli, L. Orsini, F. Pantaleo, L. Pape, E. Perez, M. Peruzzi, A. Petrilli, G. Petrucciani, A. Pfeiffer, M. Pierini, F. M. Pitters, D. Rabady, A. Racz, T. Reis, M. Rovere, H. Sakulin, C. Schäfer, C. Schwick, M. Seidel, M. Selvaggi, A. Sharma, P. Silva, P. Sphicas, A. Stakia, J. Steggemann, D. Treille, A. Tsirou, V. Veckalns, M. Verzetti, W. D. Zeuner, L. Caminada, K. Deiters, W. Erdmann, R. Horisberger, Q. Ingram, H. C. Kaestli, D. Kotlinski, U. Langenegger, T. Rohe, S. A. Wiederkehr, M. Backhaus, L. Bäni, P. Berger, N. Chernyavskaya, G. Dissertori, M. Dittmar, M. Donegà, C. Dorfer, T. A. Gómez Espinosa, C. Grab, D. Hits, T. Klijnsma, W. Lustermann, R. A. Manzoni, M. Marionneau, M. T. Meinhard, F. Micheli, P. Musella, F. Nessi-Tedaldi, J. Pata, F. Pauss, G. Perrin, L. Perrozzi, S. Pigazzini, M. Quittnat, C. Reissel, D. Ruini, D. A. Sanz Becerra, M. Schönenberger, L. Shchutska, V. R. Tavolaro, K. Theofilatos, M. L. Vesterbacka Olsson, R. Wallny, D. H. Zhu, T. K. Aarrestad, C. Amsler, D. Brzhechko, M. F. Canelli, A. De Cosa, R. Del Burgo, S. Donato, C. Galloni, T. Hreus, B. Kilminster, S. Leontsinis, I. Neutelings, G. Rauco, P. Robmann, D. Salerno, K. Schweiger, C. Seitz, Y. Takahashi, A. Zucchetta, Y. H. Chang, K. y. Cheng, T. H. Doan, R. Khurana, C. M. Kuo, W. Lin, A. Pozdnyakov, S. S. Yu, P. Chang, Y. Chao, K. F. Chen, P. H. Chen, W.-S. Hou, Arun Kumar, Y. F. Liu, R.-S. Lu, E. Paganis, A. Psallidas, A. Steen, B. Asavapibhop, N. Srimanobhas, N. Suwonjandee, M. N. Bakirci, A. Bat, F. Boran, S. Damarseckin, Z. S. Demiroglu, F. Dolek, C. Dozen, S. Girgis, G. Gokbulut, Y. Guler, E. Gurpinar, I. Hos, C. Isik, E. E. Kangal, O. Kara, A. Kayis Topaksu, U. Kiminsu, M. Oglakci, G. Onengut, K. Ozdemir, S. Ozturk, D. Sunar Cerci, B. Tali, U. G. Tok, H. Topakli, S. Turkcapar, I. S. Zorbakir, C. Zorbilmez, B. Isildak, G. Karapinar, M. Yalvac, M. Zeyrek, I. O. Atakisi, E. Gülmez, M. Kaya, O. Kaya, S. Ozkorucuklu, S. Tekten, E. A. Yetkin, M. N. Agaras, A. Cakir, K. Cankocak, Y. Komurcu, S. Sen, B. Grynyov, L. Levchuk, F. Ball, L. Beck, J. J. Brooke, D. Burns, E. Clement, D. Cussans, O. Davignon, H. Flacher, J. Goldstein, G. P. Heath, H. F. Heath, L. Kreczko, D. M. Newbold, S. Paramesvaran, B. Penning, T. Sakuma, D. Smith, V. J. Smith, J. Taylor, A. Titterton, K. W. Bell, A. Belyaev, C. Brew, R. M. Brown, D. Cieri, D. J. A. Cockerill, J. A. Coughlan, K. Harder, S. Harper, J. Linacre, E. Olaiya, D. Petyt, C. H. Shepherd-Themistocleous, A. Thea, I. R. Tomalin, T. Williams, W. J. Womersley, R. Bainbridge, P. Bloch, J. Borg, S. Breeze, O. Buchmuller, A. Bundock, D. Colling, P. Dauncey, G. Davies, M. Della Negra, R. Di Maria, G. Hall, G. Iles, T. James, M. Komm, C. Laner, L. Lyons, A.-M. Magnan, S. Malik, A. Martelli, J. Nash, A. Nikitenko, V. Palladino, M. Pesaresi, D. M. Raymond, A. Richards, A. Rose, E. Scott, C. Seez, A. Shtipliyski, G. Singh, M. Stoye, T. Strebler, S. Summers, A. Tapper, K. Uchida, T. Virdee, N. Wardle, D. Winterbottom, J. Wright, S. C. Zenz, J. E. Cole, P. R. Hobson, A. Khan, P. Kyberd, C. K. Mackay, A. Morton, I. D. Reid, L. Teodorescu, S. Zahid, K. Call, J. Dittmann, K. Hatakeyama, H. Liu, C. Madrid, B. McMaster, N. Pastika, C. Smith, R. Bartek, A. Dominguez, A. Buccilli, S. I. Cooper, C. Henderson, P. Rumerio, C. West, D. Arcaro, T. Bose, D. Gastler, D. Pinna, D. Rankin, C. Richardson, J. Rohlf, L. Sulak, D. Zou, G. Benelli, X. Coubez, D. Cutts, M. Hadley, J. Hakala, U. Heintz, J. M. Hogan, K. H. M. Kwok, E. Laird, G. Landsberg, J. Lee, Z. Mao, M. Narain, S. Sagir, R. Syarif, E. Usai, D. Yu, R. Band, C. Brainerd, R. Breedon, D. Burns, M. Calderon De La Barca Sanchez, M. Chertok, J. Conway, R. Conway, P. T. Cox, R. Erbacher, C. Flores, G. Funk, W. Ko, O. Kukral, R. Lander, M. Mulhearn, D. Pellett, J. Pilot, S. Shalhout, M. Shi, D. Stolp, D. Taylor, K. Tos, M. Tripathi, Z. Wang, F. Zhang, M. Bachtis, C. Bravo, R. Cousins, A. Dasgupta, A. Florent, J. Hauser, M. Ignatenko, N. Mccoll, S. Regnard, D. Saltzberg, C. Schnaible, V. Valuev, E. Bouvier, K. Burt, R. Clare, J. W. Gary, S. M. A. Ghiasi Shirazi, G. Hanson, G. Karapostoli, E. Kennedy, F. Lacroix, O. R. Long, M. Olmedo Negrete, M. I. Paneva, W. Si, L. Wang, H. Wei, S. Wimpenny, B. R. Yates, J. G. Branson, P. Chang, S. Cittolin, M. Derdzinski, R. Gerosa, D. Gilbert, B. Hashemi, A. Holzner, D. Klein, G. Kole, V. Krutelyov, J. Letts, M. Masciovecchio, D. Olivito, S. Padhi, M. Pieri, M. Sani, V. Sharma, S. Simon, M. Tadel, A. Vartak, S. Wasserbaech, J. Wood, F. Würthwein, A. Yagil, G. Zevi Della Porta, N. Amin, R. Bhandari, J. Bradmiller-Feld, C. Campagnari, M. Citron, A. Dishaw, V. Dutta, M. Franco Sevilla, L. Gouskos, R. Heller, J. Incandela, A. Ovcharova, H. Qu, J. Richman, D. Stuart, I. Suarez, S. Wang, J. Yoo, D. Anderson, A. Bornheim, J. M. Lawhorn, N. Lu, H. B. Newman, T. Q. Nguyen, M. Spiropulu, J. R. Vlimant, R. Wilkinson, S. Xie, Z. Zhang, R. Y. Zhu, M. B. Andrews, T. Ferguson, T. Mudholkar, M. Paulini, M. Sun, I. Vorobiev, M. Weinberg, J. P. Cumalat, W. T. Ford, F. Jensen, A. Johnson, M. Krohn, E. MacDonald, T. Mulholland, R. Patel, A. Perloff, K. Stenson, K. A. Ulmer, S. R. Wagner, J. Alexander, J. Chaves, Y. Cheng, J. Chu, A. Datta, K. Mcdermott, N. Mirman, J. R. Patterson, D. Quach, A. Rinkevicius, A. Ryd, L. Skinnari, L. Soffi, S. M. Tan, Z. Tao, J. Thom, J. Tucker, P. Wittich, M. Zientek, S. Abdullin, M. Albrow, M. Alyari, G. Apollinari, A. Apresyan, A. Apyan, S. Banerjee, L. A. T. Bauerdick, A. Beretvas, J. Berryhill, P. C. Bhat, K. Burkett, J. N. Butler, A. Canepa, G. B. Cerati, H. W. K. Cheung, F. Chlebana, M. Cremonesi, J. Duarte, V. D. Elvira, J. Freeman, Z. Gecse, E. Gottschalk, L. Gray, D. Green, S. Grünendahl, O. Gutsche, J. Hanlon, R. M. Harris, S. Hasegawa, J. Hirschauer, Z. Hu, B. Jayatilaka, S. Jindariani, M. Johnson, U. Joshi, B. Klima, M. J. Kortelainen, B. Kreis, S. Lammel, D. Lincoln, R. Lipton, M. Liu, T. Liu, J. Lykken, K. Maeshima, J. M. Marraffino, D. Mason, P. McBride, P. Merkel, S. Mrenna, S. Nahn, V. O’Dell, K. Pedro, C. Pena, O. Prokofyev, G. Rakness, L. Ristori, A. Savoy-Navarro, B. Schneider, E. Sexton-Kennedy, A. Soha, W. J. Spalding, L. Spiegel, S. Stoynev, J. Strait, N. Strobbe, L. Taylor, S. Tkaczyk, N. V. Tran, L. Uplegger, E. W. Vaandering, C. Vernieri, M. Verzocchi, R. Vidal, M. Wang, H. A. Weber, A. Whitbeck, D. Acosta, P. Avery, P. Bortignon, D. Bourilkov, A. Brinkerhoff, L. Cadamuro, A. Carnes, D. Curry, R. D. Field, S. V. Gleyzer, B. M. Joshi, J. Konigsberg, A. Korytov, K. H. Lo, P. Ma, K. Matchev, H. Mei, G. Mitselmakher, D. Rosenzweig, K. Shi, D. Sperka, J. Wang, S. Wang, X. Zuo, Y. R. Joshi, S. Linn, A. Ackert, T. Adams, A. Askew, S. Hagopian, V. Hagopian, K. F. Johnson, T. Kolberg, G. Martinez, T. Perry, H. Prosper, A. Saha, C. Schiber, R. Yohay, M. M. Baarmand, V. Bhopatkar, S. Colafranceschi, M. Hohlmann, D. Noonan, M. Rahmani, T. Roy, F. Yumiceva, M. R. Adams, L. Apanasevich, D. Berry, R. R. Betts, R. Cavanaugh, X. Chen, S. Dittmer, O. Evdokimov, C. E. Gerber, D. A. Hangal, D. J. Hofman, K. Jung, J. Kamin, C. Mills, I. D. Sandoval Gonzalez, M. B. Tonjes, H. Trauger, N. Varelas, H. Wang, X. Wang, Z. Wu, J. Zhang, M. Alhusseini, B. Bilki, W. Clarida, K. Dilsiz, S. Durgut, R. P. Gandrajula, M. Haytmyradov, V. Khristenko, J.-P. Merlo, A. Mestvirishvili, A. Moeller, J. Nachtman, H. Ogul, Y. Onel, F. Ozok, A. Penzo, C. Snyder, E. Tiras, J. Wetzel, B. Blumenfeld, A. Cocoros, N. Eminizer, D. Fehling, L. Feng, A. V. Gritsan, W. T. Hung, P. Maksimovic, J. Roskes, U. Sarica, M. Swartz, M. Xiao, C. You, A. Al-bataineh, P. Baringer, A. Bean, S. Boren, J. Bowen, A. Bylinkin, J. Castle, S. Khalil, A. Kropivnitskaya, D. Majumder, W. Mcbrayer, M. Murray, C. Rogan, S. Sanders, E. Schmitz, J. D. Tapia Takaki, Q. Wang, S. Duric, A. Ivanov, K. Kaadze, D. Kim, Y. Maravin, D. R. Mendis, T. Mitchell, A. Modak, A. Mohammadi, L. K. Saini, N. Skhirtladze, F. Rebassoo, D. Wright, A. Baden, O. Baron, A. Belloni, S. C. Eno, Y. Feng, C. Ferraioli, N. J. Hadley, S. Jabeen, G. Y. Jeng, R. G. Kellogg, J. Kunkle, A. C. Mignerey, S. Nabili, F. Ricci-Tam, Y. H. Shin, A. Skuja, S. C. Tonwar, K. Wong, D. Abercrombie, B. Allen, V. Azzolini, A. Baty, G. Bauer, R. Bi, S. Brandt, W. Busza, I. A. Cali, M. D’Alfonso, Z. Demiragli, G. Gomez Ceballos, M. Goncharov, P. Harris, D. Hsu, M. Hu, Y. Iiyama, G. M. Innocenti, M. Klute, D. Kovalskyi, Y.-J. Lee, P. D. Luckey, B. Maier, A. C. Marini, C. Mcginn, C. Mironov, S. Narayanan, X. Niu, C. Paus, C. Roland, G. Roland, G. S. F. Stephans, K. Sumorok, K. Tatar, D. Velicanu, J. Wang, T. W. Wang, B. Wyslouch, S. Zhaozhong, A. C. Benvenuti, R. M. Chatterjee, A. Evans, P. Hansen, J. Hiltbrand, Sh. Jain, S. Kalafut, Y. Kubota, Z. Lesko, J. Mans, N. Ruckstuhl, R. Rusack, M. A. Wadud, J. G. Acosta, S. Oliveros, E. Avdeeva, K. Bloom, D. R. Claes, C. Fangmeier, F. Golf, R. Gonzalez Suarez, R. Kamalieddin, I. Kravchenko, J. Monroy, J. E. Siado, G. R. Snow, B. Stieger, A. Godshalk, C. Harrington, I. Iashvili, A. Kharchilava, C. Mclean, D. Nguyen, A. Parker, S. Rappoccio, B. Roozbahani, G. Alverson, E. Barberis, C. Freer, Y. Haddad, A. Hortiangtham, D. M. Morse, T. Orimoto, R. Teixeira De Lima, T. Wamorkar, B. Wang, A. Wisecarver, D. Wood, S. Bhattacharya, J. Bueghly, O. Charaf, K. A. Hahn, N. Mucia, N. Odell, M. H. Schmitt, K. Sung, M. Trovato, M. Velasco, R. Bucci, N. Dev, M. Hildreth, K. Hurtado Anampa, C. Jessop, D. J. Karmgard, N. Kellams, K. Lannon, W. Li, N. Loukas, N. Marinelli, F. Meng, C. Mueller, Y. Musienko, M. Planer, A. Reinsvold, R. Ruchti, P. Siddireddy, G. Smith, S. Taroni, M. Wayne, A. Wightman, M. Wolf, A. Woodard, J. Alimena, L. Antonelli, B. Bylsma, L. S. Durkin, S. Flowers, B. Francis, C. Hill, W. Ji, T. Y. Ling, W. Luo, B. L. Winer, S. Cooperstein, P. Elmer, J. Hardenbrook, S. Higginbotham, A. Kalogeropoulos, D. Lange, M. T. Lucchini, J. Luo, D. Marlow, K. Mei, I. Ojalvo, J. Olsen, C. Palmer, P. Piroué, J. Salfeld-Nebgen, D. Stickland, C. Tully, S. Malik, S. Norberg, A. Barker, V. E. Barnes, S. Das, L. Gutay, M. Jones, A. W. Jung, A. Khatiwada, B. Mahakud, D. H. Miller, N. Neumeister, C. C. Peng, S. Piperov, H. Qiu, J. F. Schulte, J. Sun, F. Wang, R. Xiao, W. Xie, T. Cheng, J. Dolen, N. Parashar, Z. Chen, K. M. Ecklund, S. Freed, F. J. M. Geurts, M. Kilpatrick, W. Li, B. P. Padley, J. Roberts, J. Rorie, W. Shi, Z. Tu, A. Zhang, A. Bodek, P. de Barbaro, R. Demina, Y. t. Duh, J. L. Dulemba, C. Fallon, T. Ferbel, M. Galanti, A. Garcia-Bellido, J. Han, O. Hindrichs, A. Khukhunaishvili, E. Ranken, P. Tan, R. Taus, A. Agapitos, J. P. Chou, Y. Gershtein, E. Halkiadakis, A. Hart, M. Heindl, E. Hughes, S. Kaplan, R. Kunnawalkam Elayavalli, S. Kyriacou, A. Lath, R. Montalvo, K. Nash, M. Osherson, H. Saka, S. Salur, S. Schnetzer, D. Sheffield, S. Somalwar, R. Stone, S. Thomas, P. Thomassen, M. Walker, A. G. Delannoy, J. Heideman, G. Riley, S. Spanier, O. Bouhali, A. Celik, M. Dalchenko, M. De Mattia, A. Delgado, S. Dildick, R. Eusebi, J. Gilmore, T. Huang, T. Kamon, S. Luo, R. Mueller, D. Overton, L. Perniè, D. Rathjens, A. Safonov, N. Akchurin, J. Damgov, F. De Guio, P. R. Dudero, S. Kunori, K. Lamichhane, S. W. Lee, T. Mengke, S. Muthumuni, T. Peltola, S. Undleeb, I. Volobouev, Z. Wang, S. Greene, A. Gurrola, R. Janjam, W. Johns, C. Maguire, A. Melo, H. Ni, K. Padeken, J. D. Ruiz Alvarez, P. Sheldon, S. Tuo, J. Velkovska, M. Verweij, Q. Xu, M. W. Arenton, P. Barria, B. Cox, R. Hirosky, M. Joyce, A. Ledovskoy, H. Li, C. Neu, T. Sinthuprasith, Y. Wang, E. Wolfe, F. Xia, R. Harr, P. E. Karchin, N. Poudyal, J. Sturdy, P. Thapa, S. Zaleski, M. Brodski, J. Buchanan, C. Caillol, D. Carlsmith, S. Dasu, I. De Bruyn, L. Dodd, B. Gomber, M. Grothe, M. Herndon, A. Hervé, U. Hussain, P. Klabbers, A. Lanaro, K. Long, R. Loveless, T. Ruggles, A. Savin, V. Sharma, N. Smith, W. H. Smith, N. Woods, N. Woods, CMS Collaboration

**Affiliations:** 1 Yerevan Physics Institute, Yerevan, Armenia; 2 Institut für Hochenergiephysik, Wien, Austria; 3 Institute for Nuclear Problems, Minsk, Belarus; 4 Universiteit Antwerpen, Antwerp, Belgium; 5 Vrije Universiteit Brussel, Brussels, Belgium; 6 Université Libre de Bruxelles, Brussels, Belgium; 7 Ghent University, Ghent, Belgium; 8 Université Catholique de Louvain, Louvain-la-Neuve, Belgium; 9 Centro Brasileiro de Pesquisas Fisicas, Rio de Janeiro, Brazil; 10 Universidade do Estado do Rio de Janeiro, Rio de Janeiro, Brazil; 11 Universidade Estadual Paulista, Universidade Federal do ABC, São Paulo, Brazil; 12 Institute for Nuclear Research and Nuclear Energy, Bulgarian Academy of Sciences, Sofia, Bulgaria; 13 University of Sofia, Sofia, Bulgaria; 14 Beihang University, Beijing, China; 15 Institute of High Energy Physics, Beijing, China; 16 State Key Laboratory of Nuclear Physics and Technology, Peking University, Beijing, China; 17 Tsinghua University, Beijing, China; 18 Universidad de Los Andes, Bogota, Colombia; 19 University of Split, Faculty of Electrical Engineering, Mechanical Engineering and Naval Architecture, Split, Croatia; 20 University of Split, Faculty of Science, Split, Croatia; 21 Institute Rudjer Boskovic, Zagreb, Croatia; 22 University of Cyprus, Nicosia, Cyprus; 23 Charles University, Prague, Czech Republic; 24 Escuela Politecnica Nacional, Quito, Ecuador; 25 Universidad San Francisco de Quito, Quito, Ecuador; 26 Academy of Scientific Research and Technology of the Arab Republic of Egypt, Egyptian Network of High Energy Physics, Cairo, Egypt; 27 National Institute of Chemical Physics and Biophysics, Tallinn, Estonia; 28 Department of Physics, University of Helsinki, Helsinki, Finland; 29 Helsinki Institute of Physics, Helsinki, Finland; 30 Lappeenranta University of Technology, Lappeenranta, Finland; 31 IRFU, CEA, Université Paris-Saclay, Gif-sur-Yvette, France; 32 Laboratoire Leprince-Ringuet, Ecole polytechnique, CNRS/IN2P3, Université Paris-Saclay, Palaiseau, France; 33 Université de Strasbourg, CNRS, IPHC UMR 7178, Strasbourg, France; 34 Centre de Calcul de l’Institut National de Physique Nucleaire et de Physique des Particules, CNRS/IN2P3, Villeurbanne, France; 35 Université de Lyon, Université Claude Bernard Lyon 1, CNRS-IN2P3, Institut de Physique Nucléaire de Lyon, Villeurbanne, France; 36 Georgian Technical University, Tbilisi, Georgia; 37 Tbilisi State University, Tbilisi, Georgia; 38 RWTH Aachen University, I. Physikalisches Institut, Aachen, Germany; 39 RWTH Aachen University, III. Physikalisches Institut A, Aachen, Germany; 40 RWTH Aachen University, III. Physikalisches Institut B, Aachen, Germany; 41 Deutsches Elektronen-Synchrotron, Hamburg, Germany; 42 University of Hamburg, Hamburg, Germany; 43 Karlsruher Institut fuer Technologie, Karlsruhe, Germany; 44 Institute of Nuclear and Particle Physics (INPP), NCSR Demokritos, Agia Paraskevi, Greece; 45 National and Kapodistrian University of Athens, Athens, Greece; 46 National Technical University of Athens, Athens, Greece; 47 University of Ioánnina, Ioannina, Greece; 48 MTA-ELTE Lendület CMS Particle and Nuclear Physics Group, Eötvös Loránd University, Budapest, Hungary; 49 Wigner Research Centre for Physics, Budapest, Hungary; 50 Institute of Nuclear Research ATOMKI, Debrecen, Hungary; 51 Institute of Physics, University of Debrecen, Debrecen, Hungary; 52 Indian Institute of Science (IISc), Bangalore, India; 53 National Institute of Science Education and Research, HBNI, Bhubaneswar, India; 54 Panjab University, Chandigarh, India; 55 University of Delhi, Delhi, India; 56 Saha Institute of Nuclear Physics, HBNI, Kolkata, India; 57 Indian Institute of Technology Madras, Madras, India; 58 Bhabha Atomic Research Centre, Mumbai, India; 59 Tata Institute of Fundamental Research-A, Mumbai, India; 60 Tata Institute of Fundamental Research-B, Mumbai, India; 61 Indian Institute of Science Education and Research (IISER), Pune, India; 62 Institute for Research in Fundamental Sciences (IPM), Tehran, Iran; 63 University College Dublin, Dublin, Ireland; 64 INFN Sezione di Bari, Università di Bari, Politecnico di Bari, Bari, Italy; 65 INFN Sezione di Bologna, Università di Bologna, Bologna, Italy; 66 INFN Sezione di Catania, Università di Catania, Catania, Italy; 67 INFN Sezione di Firenze, Università di Firenze, Florence, Italy; 68 INFN Laboratori Nazionali di Frascati, Frascati, Italy; 69 INFN Sezione di Genova, Università di Genova, Genoa, Italy; 70 INFN Sezione di Milano-Bicocca, Università di Milano-Bicocca, Milan, Italy; 71 INFN Sezione di Napoli, Università di Napoli ’Federico II’ , Napoli, Italy, Università della Basilicata, Potenza, Italy, Università G. Marconi, Rome, Italy; 72 INFN Sezione di Padova, Università di Padova, Padova, Italy, Università di Trento, Trento, Italy; 73 INFN Sezione di Pavia, Università di Pavia, Pavia, Italy; 74 INFN Sezione di Perugia, Università di Perugia, Perugia, Italy; 75 INFN Sezione di Pisa, Università di Pisa, Scuola Normale Superiore di Pisa, Pisa, Italy; 76 INFN Sezione di Roma, Sapienza Università di Roma, Rome, Italy; 77 INFN Sezione di Torino, Università di Torino, Torino, Italy, Università del Piemonte Orientale, Novara, Italy; 78 INFN Sezione di Trieste, Università di Trieste, Trieste, Italy; 79 Kyungpook National University, Daegu, Korea; 80 Chonnam National University, Institute for Universe and Elementary Particles, Kwangju, Korea; 81 Hanyang University, Seoul, Korea; 82 Korea University, Seoul, Korea; 83 Sejong University, Seoul, Korea; 84 Seoul National University, Seoul, Korea; 85 University of Seoul, Seoul, Korea; 86 Sungkyunkwan University, Suwon, Korea; 87 Vilnius University, Vilnius, Lithuania; 88 National Centre for Particle Physics, Universiti Malaya, Kuala Lumpur, Malaysia; 89 Universidad de Sonora (UNISON), Hermosillo, Mexico; 90 Centro de Investigacion y de Estudios Avanzados del IPN, Mexico City, Mexico; 91 Universidad Iberoamericana, Mexico City, Mexico; 92 Benemerita Universidad Autonoma de Puebla, Puebla, Mexico; 93 Universidad Autónoma de San Luis Potosí, San Luis Potosí, Mexico; 94 University of Auckland, Auckland, New Zealand; 95 University of Canterbury, Christchurch, New Zealand; 96 National Centre for Physics, Quaid-I-Azam University, Islamabad, Pakistan; 97 National Centre for Nuclear Research, Swierk, Poland; 98 Institute of Experimental Physics, Faculty of Physics, University of Warsaw, Warsaw, Poland; 99 Laboratório de Instrumentação e Física Experimental de Partículas, Lisbon, Portugal; 100 Joint Institute for Nuclear Research, Dubna, Russia; 101 Petersburg Nuclear Physics Institute, Gatchina (St. Petersburg), Russia; 102 Institute for Nuclear Research, Moscow, Russia; 103 Institute for Theoretical and Experimental Physics, Moscow, Russia; 104 Moscow Institute of Physics and Technology, Moscow, Russia; 105 National Research Nuclear University ’Moscow Engineering Physics Institute’ (MEPhI), Moscow, Russia; 106 P.N. Lebedev Physical Institute, Moscow, Russia; 107 Skobeltsyn Institute of Nuclear Physics, Lomonosov Moscow State University, Moscow, Russia; 108 Novosibirsk State University (NSU), Novosibirsk, Russia; 109 Institute for High Energy Physics of National Research Centre ’Kurchatov Institute’, Protvino, Russia; 110 National Research Tomsk Polytechnic University, Tomsk, Russia; 111 University of Belgrade, Faculty of Physics and Vinca Institute of Nuclear Sciences, Belgrade, Serbia; 112 Centro de Investigaciones Energéticas Medioambientales y Tecnológicas (CIEMAT), Madrid, Spain; 113 Universidad Autónoma de Madrid, Madrid, Spain; 114 Universidad de Oviedo, Oviedo, Spain; 115 Instituto de Física de Cantabria (IFCA), CSIC-Universidad de Cantabria, Santander, Spain; 116 Department of Physics, University of Ruhuna, Matara, Sri Lanka; 117 CERN, European Organization for Nuclear Research, Geneva, Switzerland; 118 Paul Scherrer Institut, Villigen, Switzerland; 119 ETH Zurich, Institute for Particle Physics and Astrophysics (IPA), Zurich, Switzerland; 120 Universität Zürich, Zurich, Switzerland; 121 National Central University, Chung-Li, Taiwan; 122 National Taiwan University (NTU), Taipei, Taiwan; 123 Chulalongkorn University, Faculty of Science, Department of Physics, Bangkok, Thailand; 124 Çukurova University, Physics Department, Science and Art Faculty, Adana, Turkey; 125 Middle East Technical University, Physics Department, Ankara, Turkey; 126 Bogazici University, Istanbul, Turkey; 127 Istanbul Technical University, Istanbul, Turkey; 128 Institute for Scintillation Materials of National Academy of Science of Ukraine, Kharkov, Ukraine; 129 National Scientific Center, Kharkov Institute of Physics and Technology, Kharkov, Ukraine; 130 University of Bristol, Bristol, UK; 131 Rutherford Appleton Laboratory, Didcot, UK; 132 Imperial College, London, UK; 133 Brunel University, Uxbridge, UK; 134 Baylor University, Waco, USA; 135 Catholic University of America, Washington, DC USA; 136 The University of Alabama, Tuscaloosa, USA; 137 Boston University, Boston, USA; 138 Brown University, Providence, USA; 139 University of California, Davis, Davis, USA; 140 University of California, Los Angeles, USA; 141 University of California, Riverside, Riverside, USA; 142 University of California, San Diego, La Jolla, USA; 143 Department of Physics, University of California, Santa Barbara, Santa Barbara, USA; 144 California Institute of Technology, Pasadena, USA; 145 Carnegie Mellon University, Pittsburgh, USA; 146 University of Colorado Boulder, Boulder, USA; 147 Cornell University, Ithaca, USA; 148 Fermi National Accelerator Laboratory, Batavia, USA; 149 University of Florida, Gainesville, USA; 150 Florida International University, Miami, USA; 151 Florida State University, Tallahassee, USA; 152 Florida Institute of Technology, Melbourne, USA; 153 University of Illinois at Chicago (UIC), Chicago, USA; 154 The University of Iowa, Iowa City, USA; 155 Johns Hopkins University, Baltimore, USA; 156 The University of Kansas, Lawrence, USA; 157 Kansas State University, Manhattan, USA; 158 Lawrence Livermore National Laboratory, Livermore, USA; 159 University of Maryland, College Park, USA; 160 Massachusetts Institute of Technology, Cambridge, USA; 161 University of Minnesota, Minneapolis, USA; 162 University of Mississippi, Oxford, USA; 163 University of Nebraska-Lincoln, Lincoln, USA; 164 State University of New York at Buffalo, Buffalo, USA; 165 Northeastern University, Boston, USA; 166 Northwestern University, Evanston, USA; 167 University of Notre Dame, Notre Dame, USA; 168 The Ohio State University, Columbus, USA; 169 Princeton University, Princeton, USA; 170 University of Puerto Rico, Mayaguez, USA; 171 Purdue University, West Lafayette, USA; 172 Purdue University Northwest, Hammond, USA; 173 Rice University, Houston, USA; 174 University of Rochester, Rochester, USA; 175 Rutgers, The State University of New Jersey, Piscataway, USA; 176 University of Tennessee, Knoxville, USA; 177 Texas A&M University, College Station, USA; 178 Texas Tech University, Lubbock, USA; 179 Vanderbilt University, Nashville, USA; 180 University of Virginia, Charlottesville, USA; 181 Wayne State University, Detroit, USA; 182 University of Wisconsin, Madison, Madison, WI USA; 183 CERN, 1211 Geneva 23, Switzerland

## Abstract

A search is presented for the single production of vector-like quarks in proton–proton collisions at $\sqrt{s}=13\,\text {TeV}$. The data, corresponding to an integrated luminosity of 35.9$\,\text {fb}^{-1}$, were recorded with the CMS experiment at the LHC. The analysis focuses on the vector-like quark decay into a top quark and a $\mathrm {W}$ boson, with one muon or electron in the final state. The mass of the vector-like quark candidate is reconstructed from hadronic jets, the lepton, and the missing transverse momentum. Methods for the identification of $\mathrm {b}$ quarks and of highly Lorentz boosted hadronically decaying top quarks and $\mathrm {W}$ bosons are exploited in this search. No significant deviation from the standard model background expectation is observed. Exclusion limits at 95% confidence level are set on the product of the production cross section and branching fraction as a function of the vector-like quark mass, which range from 0.3 to 0.03$\,\text {pb}$ for vector-like quark masses of 700 to 2000$\,\text {GeV}$. Mass exclusion limits up to 1660$\,\text {GeV}$ are obtained, depending on the vector-like quark type, coupling, and decay width. These represent the most stringent exclusion limits for the single production of vector-like quarks in this channel.

## Introduction

The discovery of the Higgs boson ($\mathrm {H}$) [1, 2] with a mass of 125$\,\text {GeV}$ completes the particle content of the standard model (SM). Even though the SM yields numerous accurate predictions, there are several open questions, among them the origin of the $\mathrm {H}$ mass stability at the electroweak scale. Various models beyond the SM have been proposed that stabilise the $\mathrm {H}$ mass at the measured value; some examples are Little Higgs [3–5] or Composite Higgs models [6], in which additional top quark partners with masses at the TeV scale are predicted. Since the left- (LH) and right-handed (RH) chiral components of these particles transform in the same way under the SM electroweak symmetry group, they are often referred to as “vector-like quarks” (VLQs). In contrast to a fourth chiral quark generation, their impact on the $\mathrm {H}$ properties is small, such that VLQs have not been excluded by the measurements of $\mathrm {H}$ mediated cross sections [7–9].

Several searches for VLQs have been performed at the CERN LHC, setting lower exclusion limits on the VLQ mass $m_\mathrm {{VLQ}}$ [10–31]. Many of these analyses study the pair production of VLQs via the strong interaction. In contrast, the analysis presented here searches for the single VLQ production via the weak interaction, where a hadronic jet is emitted at a low angle with respect to the beam direction. Furthermore, VLQs with enhanced couplings to the third generation quarks (i.e. VLQ $\mathrm {B}$ and $X_{5/3}$ quarks with an electric charge of 1 / 3 and 5 / 3 respectively) are produced in association with a bottom ($\mathrm {b}$) or top ($\mathrm {t}$) quark, leading to the $\mathrm {B}$ +$\mathrm {b}$, $\mathrm {B}$ +$\mathrm {t}$, and $X_{5/3}$+$\mathrm {t}$ production modes.

**Fig. 1:**
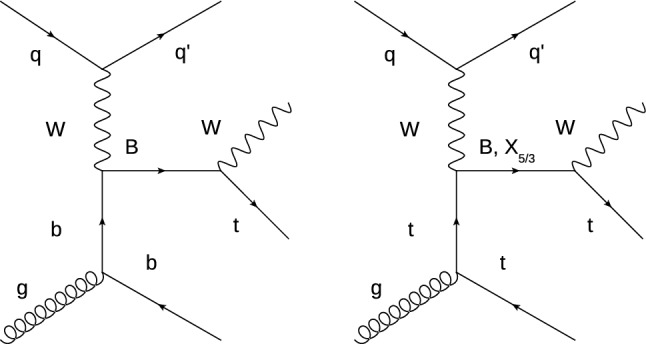
Leading order Feynman diagrams for the production of a single vector-like $\mathrm {B}$ or $X_{5/3}$ quark in association with a $\mathrm {b}$ (left) or $\mathrm {t}$ (right) and a light-flavour quark, and the subsequent decay of the VLQ to $\mathrm {t}\mathrm {W}$

While a VLQ $\mathrm {B}$ quark could decay into the $\mathrm {H} \mathrm {b}$, $\mathrm {Z} \mathrm {b}$, or $\mathrm {t}\mathrm {W}$ final state, a VLQ $X_{5/3}$ quark could only decay into the $\mathrm {t}$ $\mathrm {W}$ final state. This search focuses on the $\mathrm {t}$ $\mathrm {W}$ final state. In Fig. 1, two leading-order (LO) Feynman diagrams are shown for the single production of $\mathrm {B}$ and $X_{5/3}$ quarks and their decay into $\mathrm {t}$ $\mathrm {W}$. This paper presents the first search of this signature in proton–proton ($\mathrm {p}\mathrm {p}$) collision data recorded at a centre-of-mass energy of 13 $\,\text {TeV}$. Results at $\sqrt{s} = 8 \,\text {TeV}$ have been obtained by the ATLAS collaboration [32].

In this analysis, final states with a single muon or electron, several hadronic jets, and missing transverse momenta $p_{\mathrm {T}} ^\text {miss}$ are studied. Because of the high mass of the VLQ, the $\mathrm {t}$ and $\mathrm {W}$ can have high Lorentz boosts, leading to highly collimated decays of the $\mathrm {W}$ boson, the top quark and non-isolated leptons. For signal events, the mass of the $\mathrm {B}$ and $X_{5/3}$ quarks can be reconstructed using hadronic jets, the lepton, and the $p_{\mathrm {T}} ^\text {miss}$. The associated $\mathrm {b}$ and $\mathrm {t}$, as well as the leptons originating from their decay, have much lower transverse momenta $p_{\mathrm {T}}$ and are not considered for the reconstruction or selection.

The dominant SM background processes are top quark pair (${\mathrm {t}\overline{\mathrm {t}}}$) production, $\mathrm {W}$+jets and $\mathrm {Z}$+jets production, single $\mathrm {t}$ production, and multijet production via the strong force. All SM backgrounds contributing to this search are predicted from dedicated control regions in data, defined through the absence of a forward jet.

This paper is organised as follows: Sect. 2 provides a description of the CMS detector. Section 3 introduces the data set and the simulated events. This is followed by the event selection in Sect. 4, as well as by the description of the reconstruction of the VLQ mass in Sect. 5. In Sect. 6, a method to estimate the background is discussed. Systematic uncertainties are detailed in Sect. 7. The final results of the analysis, as well as the statistical interpretation in terms of exclusion limits, are discussed in Sect. 8.

## The CMS detector and physics objects

The central feature of the CMS apparatus is a superconducting solenoid of 6$\,\text {m}$ internal diameter, providing a magnetic field of 3.8$\,\text {T}$. Within the solenoid volume are a silicon pixel and strip tracker, a lead tungstate crystal electromagnetic calorimeter (ECAL), and a brass and scintillator hadron calorimeter (HCAL), each composed of a barrel and two endcap sections. Forward calorimeters extend the pseudorapidity coverage provided by the barrel and endcap detectors. Muons are detected in gas-ionisation chambers embedded in the steel flux-return yoke outside the solenoid.

The particle-flow event algorithm [33] aims to reconstruct and identify each individual particle with an optimised combination of information from the various elements of the CMS detector. The energy of photons is directly obtained from the ECAL measurement, corrected for zero-suppression effects. The energy of electrons is determined from a combination of the electron momentum at the primary interaction vertex, the energy of the corresponding ECAL cluster, and the energy sum of all bremsstrahlung photons spatially compatible with originating from the electron track [34]. The energy of muons is obtained from the curvature of the corresponding track [35]. The energy of charged hadrons is determined from a combination of their momentum measured in the tracker and the matching ECAL and HCAL energy deposits, corrected for zero-suppression effects and for the response function of the calorimeters to hadronic showers. Finally, the energy of neutral hadrons is obtained from the corresponding corrected ECAL and HCAL energy.

The reconstructed vertex with the largest value of summed physics-object $p_{\mathrm {T}} ^2$ is taken to be the primary $\mathrm {p}\mathrm {p}$ interaction vertex. The physics objects used are the jets, clustered with the jet finding algorithm [36, 37] with the tracks assigned to the vertex as inputs, and the associated missing transverse momentum, taken as the negative vector sum of the $p_{\mathrm {T}}$ of those jets.

A more detailed description of the CMS detector, together with a definition of the coordinate system used and the relevant kinematic variables, can be found in Ref. [38].

## Data and simulated samples

In this analysis, $\mathrm {p}\mathrm {p}$ collision data at a centre-of-mass energy of $13\,\text {TeV}$ taken in 2016 by the CMS experiment are analyzed. The data have been collected with muon and electron triggers [39]. For the muon trigger, a muon candidate with $p_{\mathrm {T}} > 50 \,\text {GeV}$ is required. Data events in the electron channel are collected using a logical combination of two triggers: the first requires an electron candidate with $p_{\mathrm {T}} > 45\,\text {GeV}$ and a hadronic jet candidate with $p_{\mathrm {T}} > 165\,\text {GeV}$, the second requires an electron candidate with $p_{\mathrm {T}} > 115\,\text {GeV}$. In the trigger selection, reconstructed leptons and jets must be in the central part of the detector, with a pseudorapidity of $|\eta | < 2.4$. No lepton isolation criteria are applied at the trigger level. The collected data correspond to an integrated luminosity of 35.9$\,\text {fb}^{-1}$ [40].

For the study of dominant SM background processes and for the validation of the background estimation, simulated samples using Monte Carlo (MC) techniques are used. The top quark pair production via the strong interaction and single top quark production in the *t*-channel, and the $\mathrm {t}\mathrm {W}$ process are generated with the next-to-leading-order (NLO) generator powheg [41–43] (version v2 is used for the first two and version v1 for the third). The event generator MadGraph 5_amc@nlo (v2.2.2) [44] at NLO is used for single top quark production in the *s*-channel. The $\mathrm {W}$+jets and $\mathrm {Z}$+jets processes are also simulated using MadGraph 5_amc@nlo (v2.2.2). The $\mathrm {W}$+jets events are generated at NLO, and the FXFX scheme [45] is used to match the parton shower emission. The $\mathrm {Z}$+jets events are produced at LO with the MLM parton matching scheme [46]. The production of quantum chromodynamics (QCD) multijet events has been simulated at LO using pythia [47]. All generated events are interfaced with pythia for the description of the parton shower and hadronisation. The parton distribution functions (PDFs) are taken from the NNPDF 3.0 [48] sets, with the precision matching that of the matrix element calculations. The underlying event tune is CUETP8M1 [49, 50], except for the simulation of top quark pairs and single top quark production in the *t*-channel, which use CUETP8M2T4 [51].

Signal events are generated at LO using MadGraph 5_amc@nlo for $\mathrm {B}$ and $X_{5/3}$ with VLQ decay widths relative to the VLQ mass of $(\Gamma /m)_{\mathrm {VLQ}} = 1$, 10, 20, and 30%. The samples with 1% relative VLQ width are simulated in steps of 100$\,\text {GeV}$ for masses between 700 and 2000$\,\text {GeV}$. Samples with 10, 20, and 30% relative VLQ widths are generated in steps of 200$\,\text {GeV}$ for masses ranging from 800 to 2000$\,\text {GeV}$, using a modified version of the model proposed in Refs. [52–54]. Separate signal samples are generated for the two main production modes, in which VLQs are produced in association either with a $\mathrm {b}$ quark or with a $\mathrm {t}$ quark, viz. $\mathrm {p}\mathrm {p}\rightarrow \mathrm {B} \mathrm {b}\mathrm {q}$ and $\mathrm {p}\mathrm {p}\rightarrow \mathrm {B} \mathrm {t}\mathrm {q}$. The theoretical cross sections for VLQ production are calculated using Refs. [55–57], where a simplified approach is used to provide a model-independent interpretation of experimental results for narrow and large mass width scenarios, as already used for the interpretation of singly produced vector-like $\mathrm {T}$ and $\mathrm {B}$ quarks [18, 19]. The $\textsc {MADSPIN}$ package [58, 59] is used to retain the correct spin correlations of the top quark and $\mathrm {W}$ boson decay products. Interference effects between signal and SM processes have been found to be negligible in this analysis.

All generated events are passed through a Geant4 [60] based detector simulation of the CMS detector. Additional $\mathrm {p}\mathrm {p}$ interactions originating from the same bunch crossing (in-time pileup), as well as from the following or previous bunch crossings (out-of-time pileup) are taken into account in the simulation.

## Event selection

The physics objects used in this analysis are muons, electrons, hadronic jets, ${\vec {p}}_{\mathrm {T}}^{\text {miss}}$, and $S_{\mathrm {T},\text {lep}}$ (defined as the scalar sum of the lepton $p_{\mathrm {T}}$ and $p_{\mathrm {T}} ^\text {miss}$).

For each event, jets are clustered from reconstructed particles using the infrared and collinear safe anti-$k_{\mathrm {T}}$ algorithm [36] with a distance parameter $R=0.4$ (AK4 jet). Additionally, jets with $R=0.8$ (AK8 jet) are also clustered in every event with the anti-$k_{\mathrm {T}}$ algorithm, which are used for $\mathrm {t}$ and $\mathrm {W}$ tagging. The jet clustering is performed with the FastJet [37] package. Jet momentum is determined as the vectorial sum of all particle momenta in the jet, and is found from simulation to be within 5–10% of the true momentum over the whole $p_{\mathrm {T}}$ spectrum and detector acceptance. Additional $\mathrm {p}\mathrm {p}$ interactions within the same or nearby bunch crossings can contribute additional tracks and calorimetric energy depositions to the jet momentum. To mitigate this effect, tracks identified to be originating from pileup vertices are discarded, and an offset correction is applied to correct for remaining contributions. Jet energy corrections are derived from simulation studies so that the average measured response of jets becomes identical to that of particle level jets. In situ measurements of the momentum balance in dijet, photon+jet, $\mathrm {Z}$+jet, and multijet events are used to account for any residual differences in the jet energy scale in data and simulation. Additional selection criteria are applied to each jet to remove jets potentially dominated by anomalous contributions from various subdetector components or reconstruction failures [61].

From the corrected and reconstructed AK4 jet$\mathrm {s}$, those are considered that have $p_{\mathrm {T}} > 30 \,\text {GeV}$ and $|\eta | < 4$, while AK8 jet$\mathrm {s}$ must have $p_{\mathrm {T}} >170 \,\text {GeV}$ and $|\eta | < 2.4$.

Events selected in the analysis are required to have one reconstructed muon or electron with $p_{\mathrm {T}} >55 \,\text {GeV}$ and $|\eta | < 2.4$. Electrons and muons are selected using tight quality criteria with small misidentification probabilities of about 0.1% for muons and 1% for electrons [34, 62]. In the electron channel, a AK4 jet must have $p_{\mathrm {T}} >185 \,\text {GeV}$ and $|\eta | < 2.4$ if the electron has $p_{\mathrm {T}} <120 \,\text {GeV}$, reflecting the trigger selection. Events with more than one muon or electron passing the same tight identification criteria and having $p_{\mathrm {T}} > 40 \,\text {GeV}$ and $|\eta | < 2.4$ are discarded. Selected events contain two AK4 jet$\mathrm {s}$ with $p_{\mathrm {T}} > 50 \,\text {GeV}$, which are in the central part of the detector with $|\eta | < 2.4$. Additionally at least one AK8 jet is required. For the reconstruction AK4 jet$\mathrm {s}$ are used with $p_{\mathrm {T}} > 30 \,\text {GeV}$ and $|\eta | < 2.4$, while the AK4 jet$\mathrm {s}$ emitted close to the beam pipe and employed in the background estimation must fulfill $p_{\mathrm {T}} > 30 \,\text {GeV}$ and $2.4< |\eta | < 4$.

Because of the high Lorentz boosts of the top quarks and $\mathrm {W}$ bosons from the heavy VLQ decay, signal events can have leptons in close vicinity to the jets. For this reason, standard lepton isolation would reduce the selection efficiency considerably. Therefore, for the suppression of events originating from QCD mulitjet processes, either the perpendicular component of the lepton momentum relative to the geometrically closest AK4 jet $p_{\mathrm {T,rel}}$, is required to exceed $40 \,\text {GeV}$ or the angular distance of the lepton to the jet, ${\varDelta }R (\ell ,\mathrm {jet}) = \sqrt{\smash [b]{({\varDelta }\eta )^2 + ({\varDelta }\phi )^2}}$, must be larger than 0.4, where $\phi$ is the azimuthal angle in radians. Furthermore, for selecting an event, the magnitude of ${\vec {p}}_{\mathrm {T}}^{\text {miss}}$ has to be greater than 50$\,\text {GeV}$ in the muon channel and greater than 60$\,\text {GeV}$ in the electron channel. This requirement reduces the amount of background from multijet production. The final selection is based on the variable $S_{\mathrm {T},\text {lep}}$, which is required to be larger than 250$\,\text {GeV}$ in the muon channel and 290$\,\text {GeV}$ in the electron channel.

Events are separated into categories exploiting the tagging techniques for boosted top quarks and $\mathrm {W}$ bosons decaying hadronically, as well as for hadronic jets originating from $\mathrm {b}$ quarks. Jets with $R=0.8$ are used to identify the hadronic decays of highly boosted top quarks and $\mathrm {W}$ bosons [63, 64]. For top quark jets $p_{\mathrm {T}} >400 \,\text {GeV}$ is required, and for $\mathrm {W}$ boson jets the requirement is $p_{\mathrm {T}} >200 \,\text {GeV}$. The “soft drop” (SD) declustering and grooming algorithm [65, 66] with $z=0.1$ and $\beta =0$ is employed to identify subjets and to remove soft and wide-angle radiation. The groomed jet mass, $m_{\mathrm {SD}}$, is used to identify top quark and $\mathrm {W}$ boson candidates. Tagged top quark candidates ($\mathrm {t}$ tagged) are required to have $105< m_{\mathrm {SD}} < 220 \,\text {GeV}$ and one of the subjets must fulfill the loose $\mathrm {b}$ tagging criterion, based on the combined secondary vertex (CSVv2) [67] algorithm. The loose criterion is defined to give a 80% efficiency of correctly identifying $\mathrm {b}$ jets, with a 10% probability of incorrectly tagging a light quark jet. Additionally, the jet must have a N-subjettiness [68, 69] ratio $\tau _3/\tau _2 < 0.5$ and its angular distance to the lepton ${\varDelta }R{(\ell ,\mathrm {t}\ \mathrm {tag})}$ must be larger than 2. Identified $\mathrm {W}$ boson candidates ($\mathrm {W}$ tag) must have $65< m_{\mathrm {SD}} < 95 \,\text {GeV}$. The medium $\mathrm {b}$ tag criterion is used on AK4 jet$\mathrm {s}$, defined to give a $60\%$ efficiency of correctly identifying $\mathrm {b}$ jets, with a $1\%$ probability of incorrectly tagging a light quark jet.

Selected events are attributed to different mutually exclusive event categories. Events containing at least one $\mathrm {t}$ tag constitute the first category (“$\mathrm {t}$ tag”). If no $\mathrm {t}$ tag is found, all events with at least one $\mathrm {W}$ tag are grouped into a second category (“$\mathrm {W}$ tag”). The remaining events are attributed to three further categories based on the multiplicity of $\mathrm {b}$ tags found in the event. We distinguish events with at least two (“${\ge }2$ $\mathrm {b}$ tag”), exactly one (“1 $\mathrm {b}$ tag”), and no $\mathrm {b}$ tag (“0 $\mathrm {b}$ tag”). These five categories are built separately in the muon and in the electron channel leading to a total of ten categories.

## Mass reconstruction

Hadronic jets, leptons, and ${\vec {p}}_{\mathrm {T}}^{\text {miss}}$ are used to reconstruct the mass of the VLQ, denoted $m_\mathrm {reco}$. In signal events, the lepton in the final state always originates from the decay of a $\mathrm {W}$ boson, either the $\mathrm {W}$ boson from the VLQ decay or the $\mathrm {W}$ boson from the top quark decay. The neutrino four-momentum can thus be reconstructed from the components of ${\vec {p}}_{\mathrm {T}}^{\text {miss}}$, the $\mathrm {W}$ mass constraint, and the assumption of massless neutrinos.

In the case when a hadronic jet with a $\mathrm {t}$ tag is found, $m_\mathrm {reco}$ is calculated from the four-momentum of the $\mathrm {t}$-tagged jet and the four-momentum of the leptonically decaying $\mathrm {W}$ boson. If several hadronic jets with $\mathrm {t}$ tags are present, the one with the largest angular distance to the reconstructed leptonic $\mathrm {W}$ boson decay is used. Once the $\mathrm {t}$-tagged jet has been selected, all overlapping AK4 jet jets in the event are removed in order to avoid double counting of energy. For the shown $m_\mathrm {reco}$ distributions these events form the $\mathrm {t}$ tag category. For events in the other categories the hadronic part of the VLQ decay is reconstructed from combinations of AK4 jet$\mathrm {s}$ with $|\eta | < 2.4$. Each possible jet assignment for the decays of the $\mathrm {W}$ boson and $\mathrm {t}$ quark is tested exploiting the following $\chi ^2$ quantity

1 $$\begin{aligned} \chi ^2&= \frac{\left( m_{\mathrm {t}}- \overline{m}_{\mathrm {t}} \right) ^2}{\sigma ^2_{\mathrm {t}}} + \frac{\left( m_{\mathrm {W} }- \overline{m}_{\mathrm {W} } \right) ^2}{\sigma ^2_{\mathrm {W} }} \nonumber \\&\quad + \frac{\left( {\varDelta }R(\mathrm {t},\mathrm {W} )-\pi \right) ^2}{\sigma ^2_{{\varDelta }R}}+ \frac{\left( p_{\mathrm {T},\mathrm {W} }/ p_{\mathrm {T},\mathrm {t}}- 1\right) ^2}{\sigma ^2_{p_{\mathrm {T}}}}. \end{aligned}$$

For each event, the jet assignment with the maximum $\chi ^2$ probability is selected. For the $\chi ^2$ quantity the $p_{\mathrm {T}}$ balance, $p_{\mathrm {T},\mathrm {W} }/ p_{\mathrm {T},\mathrm {t}}$, the angular distance, ${\varDelta }R(\mathrm {t},\mathrm {W} )$, and the reconstructed masses of the top quark candidate $m_{\mathrm {t}}$ and the $\mathrm {W}$ boson candidate $m_{\mathrm {W} }$ are used. The expected values $\overline{m}_{\mathrm {t}}$ and $\overline{m}_{\mathrm {W} }$, and their standard deviations $\sigma _{\mathrm {t}}$ and $\sigma _{\mathrm {W} }$ are obtained from simulation for correctly reconstructed events and it is verified that the values are independent of the VLQ mass. Here, correctly reconstructed events are defined by the assignment of jets to generated $\mathrm {t}$ quarks and $\mathrm {W}$ bosons, where the generated particles from the VLQ decay are unambiguously matched within a distance of ${\varDelta }R < 0.4$ to the reconstructed particles. It was also verified in simulation that the expected values of ${\varDelta }R(\mathrm {t},\mathrm {W} )$ and the $p_{\mathrm {T}}$ balance are $\pi$ and 1, with their standard deviations $\sigma _{{\varDelta }R}$ and $\sigma _{p_{\mathrm {T}}}$. In order to account for cases where the $\mathrm {W}$ boson from the VLQ decay decays into a lepton and neutrino, the $\chi ^2$ is calculated for each permutation with the second term omitted. Cases where the hadronic decay products of the $\mathrm {W}$ bosons or the top quark are reconstructed in a single AK4 jet are included by omitting the first or second term in the calculation of the $\chi ^2$.

**Fig. 2:**
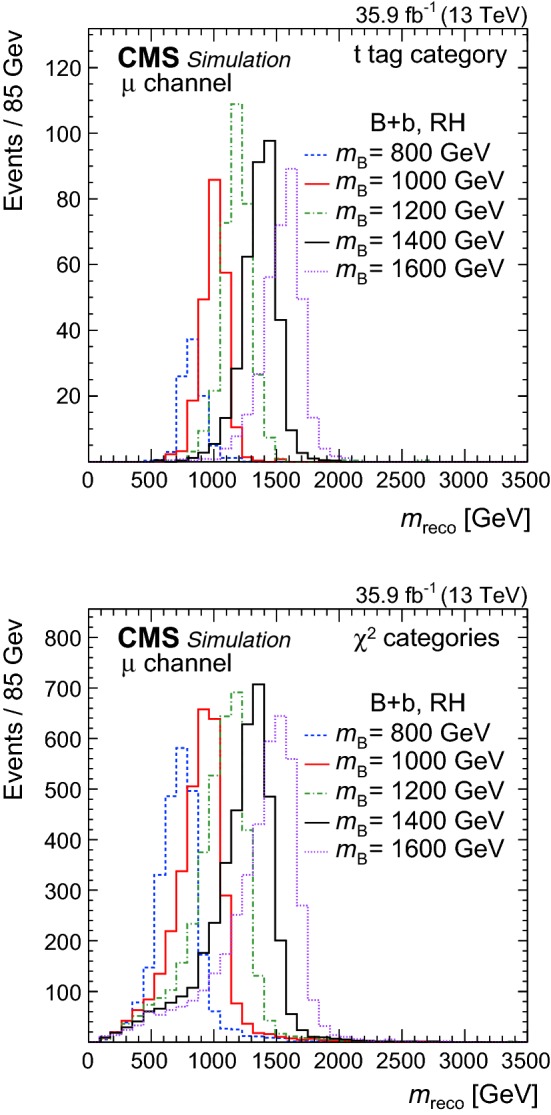
Distributions of $m_\mathrm {reco}$ for the $\mathrm {B}$ +$\mathrm {b}$ production mode, obtained for simulated events with a muon in the final state, reconstructed with a $\mathrm {t}$ tag (top) and with the $\chi ^2$ method (bottom) for right-handed VLQ couplings and various VLQ masses $m_{\mathrm {B}}$. Signal events are shown assuming a production cross section of 1$\,\text {pb}$ and a relative VLQ decay width of 1%

The distributions of $m_\mathrm {reco}$ in simulation for the $\mathrm {B}$ +$\mathrm {b}$ production mode with right-handed couplings are shown in Fig. 2 for events with a muon in the final state. The reconstruction of events with a $\mathrm {t}$ tag (top) is best suited for high VLQ masses where the decay products of the top quark are highly boosted, while the $\chi ^2$ method (bottom) yields a stable performance for all VLQ masses, where the decay products of the $\mathrm {W}$ boson and top quark are reconstructed from several jets. Additionally, the latter method enables the reconstruction of events with a lepton from the top quark decay chain. Mass resolutions between 10–15% are achieved for both reconstruction methods, with peak values of the $m_\mathrm {reco}$ distributions at the expected values. The VLQs with left-handed couplings (not shown) have a lower selection efficiency by 20–25% because of a smaller lepton $p_{\mathrm {T}}$, on average, but otherwise features a behaviour similar to VLQs with right-handed couplings. Distributions obtained for the final states with an electron are similar to those with a muon.

## Background estimation

The data sample obtained after the selection is then divided into a signal region with a jet in the forward region of the detector with $2.4< |\eta | < 4.0$ and a control region without such a jet. The distribution of background processes in the signal region is estimated using the shape of the $m_\mathrm {reco}$ distribution in the control region. Residual differences in the shapes of the $m_\mathrm {reco}$ distributions between signal and control regions are investigated in each of the signal categories by using simulated SM events. Differences can arise from different background compositions in signal and control regions due to the presence of a forward jet. The observed differences are small, with average values of 10%, and are corrected for by multiplicative factors applied to the background predictions in the validation and signal regions. The largest differences are observed for $m_\mathrm {reco}$ values below 800$\,\text {GeV}$, with values no larger than about 20%.

**Fig. 3:**
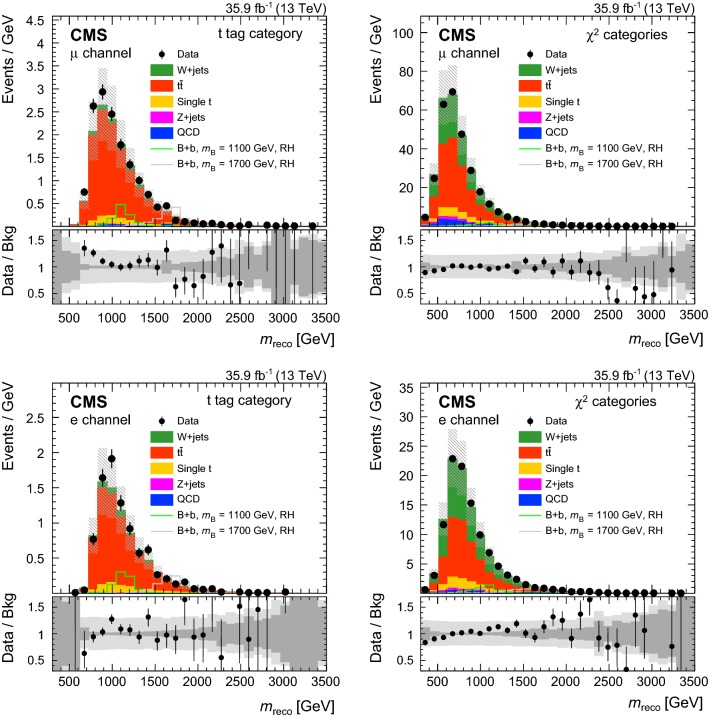
Distributions of $m_\mathrm {reco}$ in data and simulation in the control region for the muon (upper) and electron (lower) channels for events reconstructed with a $\mathrm {t}$ tag (left) and with the $\chi ^2$ method (right). The VLQ signal is shown for the $\mathrm {B}$ +$\mathrm {b}$ production mode and right-handed VLQ couplings. The vertical bars illustrate the statistical uncertainties on the data, while the shaded area shows the total uncertainties for the background simulation. The lower panels show the ratio of data to the background prediction. The dark and light gray bands correspond to the statistical and total uncertainties, respectively

In order to validate the VLQ mass reconstruction, data are compared to simulation in the control region. In Fig. 3 the distributions of $m_\mathrm {reco}$ are shown in the muon (upper) and electron (lower) channels for events with a $\mathrm {t}$ tag (left) and events reconstructed with the $\chi ^2$ method (right). The ${\mathrm {t}\overline{\mathrm {t}}}$ and $\mathrm {t}\mathrm {W}$ standard model processes provide irreducible backgrounds in the reconstructed VLQ mass distributions, showing good agreement between the data and simulation. The contribution of signal events in the control region is small and is taken into account by a simultaneous fit to signal and control regions in the statistical extraction of the results.

**Fig. 4:**
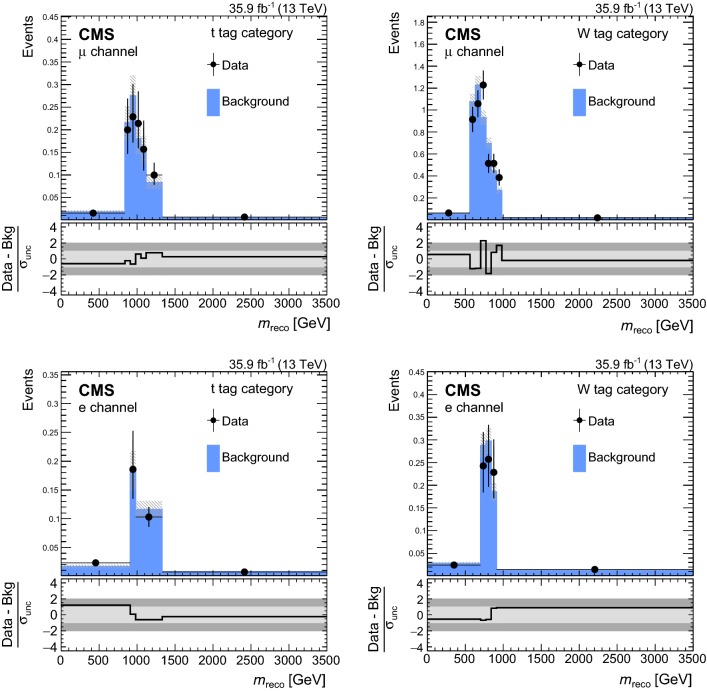
Distributions of $m_\mathrm {reco}$ in the validation region of the two most sensitive categories in the muon channel (upper) and electron channel (lower). The lower panels show the difference of data and background expectations in units of the total (stat. and sys.) uncertainty on the background estimate

In order to validate the background estimation, a validation region is constructed from requiring events with reconstruction *p*-values smaller than 0.08. The *p*-values are calculated as the probability of obtaining the $\chi ^2$ as given by Eq. (1), where the number of degrees of freedom of the selected hypothesis are taken into account. For events with a $\mathrm {t}$ tag, the same $\chi ^2$ quantity is evaluated for the selected hypothesis. The validation region has an order of magnitude fewer events than the signal region and a negligible amount of signal contamination. The $m_\mathrm {reco}$ distributions for the two most sensitive categories are shown in Fig. 4 for the muon (upper) and electron (lower) channels. The observed number of events is found to be in good agreement with the predicted number of events from the background estimation in the validation region, with no statistically significant deviations. Similar observations are made for the other signal categories.

## Systematic uncertainties

Systematic uncertainties can affect both the overall normalisation of background components and the shapes of the $m_\mathrm {reco}$ distributions for signal and background processes. The main uncertainty in the shape of the $m_\mathrm {reco}$ distribution from the background estimation based on a control region in data is related to the kinematic difference between the signal and control regions. Correction factors are applied to account for this difference, obtained from SM simulations. These uncertainties have a size of 10% on average, with maximum values of 20% at small values of $m_\mathrm {reco}$. Compared to these uncertainties, the effects from uncertainties in the SM simulations are negligible on the background estimation, as these cancel to a large degree when building the ratios between signal and control regions. The uncertainties in the overall normalisation of the background predictions are obtained from a fit to the data in the signal region.

Uncertainties in the MC simulation are applied to all simulated signal events. In the following, the systematic uncertainties are summarized.

- The uncertainty in the integrated luminosity measurement recorded with the CMS detector in the 2016 run at $\sqrt{s}=13\,\text {TeV}$ is 2.5% [40].
- The estimation of pileup effects is based on the total inelastic cross section. This cross section is determined to be 69.2$\,\text {mb}$. The uncertainty is taken into account by varying the total inelastic cross section by 4.6% [70].
- Simulated events are corrected for lepton identification, trigger, and isolation efficiencies. The corresponding corrections are applied as functions of $|\eta |$ and $p_{\mathrm {T}}$. The systematic uncertainties due to these corrections are taken into account by varying each correction factor within its uncertainty.
- The scale factors for the jet energy scale and resolution are determined as functions of $|\eta |$ and $p_{\mathrm {T}}$ [61]. The effect of the uncertainties in these scale factors are considered by varying the scale factors within their uncertainties. Jets with distance parameters of 0.4 and 0.8 are modified simultaneously. The results of variations for AK4 jet$\mathrm {s}$ are propagated to the measurement of ${\vec {p}}_{\mathrm {T}}^{\text {miss}}$.
- The uncertainties due to the PDFs are evaluated by considering 100 replicas of the NNPDF 3.0 set according to the procedure described in Ref. [71]. The associated PDF uncertainties in the signal acceptance are estimated following the prescription for the LHC [71].
- Uncertainties associated with variations of the factorisation $\mu _\mathrm {f}$ and renormalisation scales $\mu _\mathrm {r}$ are evaluated by varying the respective scales independently, by factors of 0.5 and 2.
- Corrections for the $\mathrm {b}$ tagging efficiencies and misidentification rates for AK4 jet$\mathrm {s}$, and subjets of AK8 jet$\mathrm {s}$ are applied. These are measured as a function of the jet $p_{\mathrm {T}}$ [67]. The corresponding uncertainties are taken into account by varying the corrections within their uncertainties for heavy- and light-flavour jets separately.
- An uncertainty on the $\mathrm {t}$ tagging efficiency of $+\,7$ and $-\,4\%$ is applied to signal events with a $\mathrm {t}$ tag [64]. The uncertainty on the $\mathrm {W}$ tagging efficiency is determined from jet mass resolution (JMR) and scale (JMS) uncertainties, which are added in quadrature. An additional JMR uncertainty is derived from the differences in the hadronisation and shower models of pythia and herwig++ [72]. The uncertainty depends on the $p_{\mathrm {T}}$ of the $\mathrm {W}$ boson; for VLQs with a mass of 700$\,\text {GeV}$ it is around 2% and for a mass of 1800$\,\text {GeV}$ it is around 6%. An uncertainty of 1% is assigned to the JMS, as obtained from studies of the jet mass in fully merged hadronic $\mathrm {W}$ boson decays.

In Table 1, a summary of the uncertainties considered for signal events is shown, where the largest uncertainties come from the jet energy scale and the jet tagging. For the uncertainties connected to the PDF, $\mu _\mathrm {f}$ and $\mu _\mathrm {r}$ only the signal acceptance and shape differences are propagated. The uncertainties with the largest impact on the analysis are the uncertainties associated with the data-driven background estimation, being more than two times larger than the jet energy scale uncertainties in the signal.

**Table 1:** Uncertainties considered for simulated signal events in the $\mathrm {B}$ +$\mathrm {b}$ production mode ($m_{\mathrm {B} {}} = 900 \,\text {GeV}$) for right-handed VLQ couplings for the $\mathrm {t}$ tag and $\mathrm {W}$ tag categories. The uncertainties in the $\mathrm {b}$ tag categories are of comparable size to those in the $\mathrm {W}$ tag category

Uncertainty		$\mathrm {t}$ tag ($\%$)	$\mathrm {W}$ tag ($\%$)
$\mathrm {W}$ tagging	Rate	–	3.3
$\mathrm {t}$ tagging	Rate	$^{+7}_{-4}$	–
Luminosity	Rate	2.5	2.5
Pileup	Shape	1–3	0.2
Lepton reconstruction	Shape	2–3	2–3
$\mathrm {b}$ tagging	Shape	2.5	2.5
Jet energy scale	Shape	2–6	1–5
Jet energy resolution	Shape	1–2	1–2
PDF	Shape	2–3	0.5
$\mu _f$ and $\mu _r$	Shape	0.3	0.2

## Results

The $m_\mathrm {reco}$ distributions in the ten categories are measured in the signal and control region, which are defined by the presence or absence of a forward jet with $|\eta | > 2.4$. For the background estimate in the signal regions, a simultaneous binned maximum likelihood fit of both regions is performed using the Theta [73] package. In these fits, the signal cross section and the background normalisations in the different signal categories are free parameters. The shapes of the $m_\mathrm {reco}$ distributions for the SM background in the signal regions are taken from the corresponding control regions. Systematic uncertainties are taken into account as additional nuisance parameters. A common nuisance parameter is used for uncertainties in the muon and electron channels if a similar effect is expected on the shape or normalisation of the $m_\mathrm {reco}$ distribution in both channels similarly. The nuisance parameters for the shape uncertainties are taken to be Gaussian distributed. For the uncertainties on the normalisation log-normal prior distributions are assumed.

**Fig. 5:**
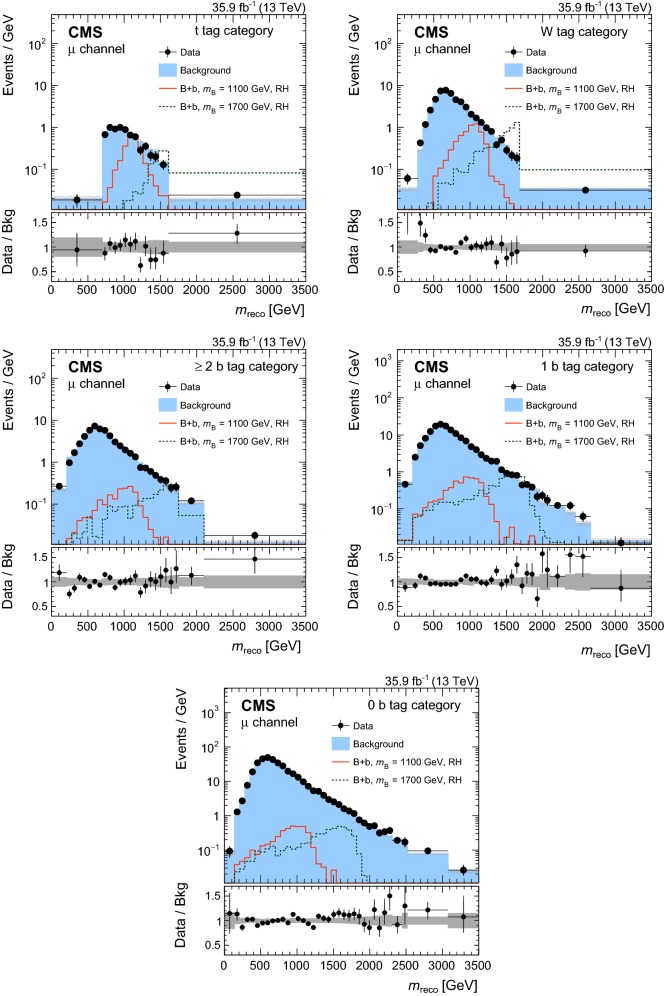
Distributions of $m_\mathrm {reco}$ measured in the signal region for events with a jet in the forward direction with $|\eta |>2.4$ in the muon channel. Shown are the sensitive categories: $\mathrm {t}$ tag (upper left), $\mathrm {W}$ tag (upper right), ${\ge }2$ $\mathrm {b}$ tag (middle left), 1 $\mathrm {b}$ tag (middle right) and 0 $\mathrm {b}$ tag (lower). The background prediction is obtained from control regions as detailed in the main text. The distributions from two example signal samples for the $\mathrm {B}$ +$\mathrm {b}$ production mode with right-handed VLQ couplings with a cross section of 1$\,\text {pb}$ and a relative width of 1% are shown for illustration

**Fig. 6:**
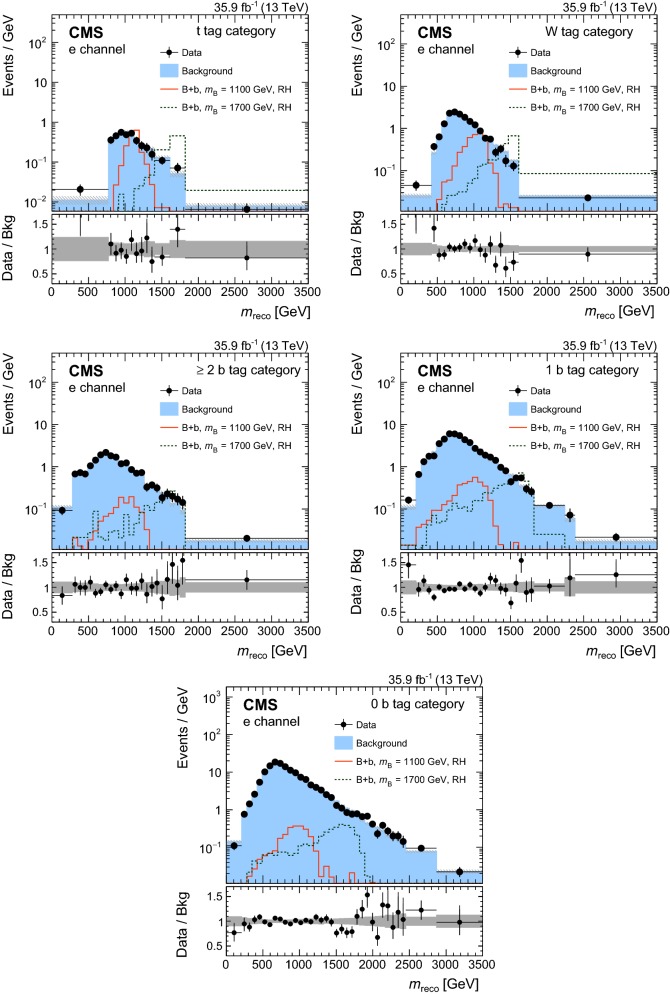
Distributions of $m_\mathrm {reco}$ measured in the signal region for events with a jet in the forward direction with $|\eta |>2.4$ in the electron channel. Shown are the sensitive categories: $\mathrm {t}$ tag(upper left), $\mathrm {W}$ tag(upper right), ${\ge }2$ $\mathrm {b}$ tag (middle left), 1 $\mathrm {b}$ tag (middle right) and 0 $\mathrm {b}$ tag (lower). The background prediction is obtained from control regions as detailed in the main text. The distributions from two example signal samples for the $\mathrm {B}$ +$\mathrm {b}$ production mode with right-handed VLQ couplings with a cross section of 1$\,\text {pb}$ and a relative VLQ width of 1% are shown for illustration

The measured distributions of $m_\mathrm {reco}$ for the signal categories are shown in Figs. 5 and 6 for the muon and electron channels, together with the background predictions obtained from the control regions. The signal $m_\mathrm {reco}$ distributions for a vector-like $\mathrm {B}$ quark with right-handed couplings produced in association with a $\mathrm {b}$ quark are shown for illustration, for two different VLQ masses with an assumed production cross section of 1$\,\text {pb}$ and a relative VLQ width of 1%. No significant deviation from the background expectation is observed in any of the categories.

Exclusion limits on the product of the VLQ production cross section and branching fraction are calculated at 95% confidence level ($\text {CL}$) for VLQ masses between 700 and 2000$\,\text {GeV}$ by using a Bayesian statistical method [73, 74]. Pseudo-experiments are performed to extract expected upper limits under the background-only hypothesis. For the signal cross section parameter an uniform prior distribution, and for the nuisance parameters log-normal prior distributions are used. The nuisance parameters are randomly varied within their ranges of validity to estimate the 68 and 95% $\text {CL}$ expected limits. Correlations between the systematic uncertainties across all channels are taken into account through a common nuisance parameter. The statistical uncertainties of the background predictions are treated as an additional Poisson nuisance parameter in each bin of the $m_\mathrm {reco}$ distribution.

**Fig. 7:**
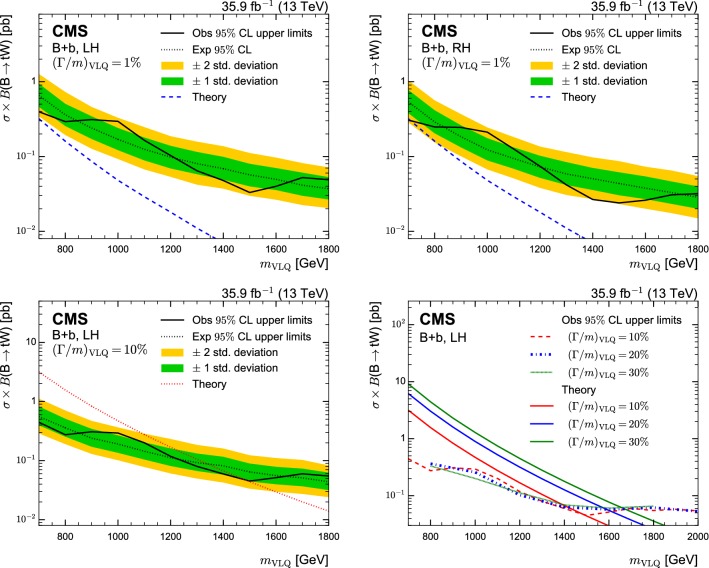
Upper limits at 95% $\text {CL}$ on the product of the VLQ production cross section and branching fraction for the $\mathrm {B}$ +$\mathrm {b}$ production mode for a relative VLQ width of 1% and left- and right-handed VLQ couplings (upper left and right), for 10% relative VLQ width and left-handed VLQ couplings (lower left), and a comparison of the observed exclusion limits for relative VLQ widths of 10, 20, and 30% for left-handed couplings (lower right). The dashed lines show the theoretical predictions

**Fig. 8:**
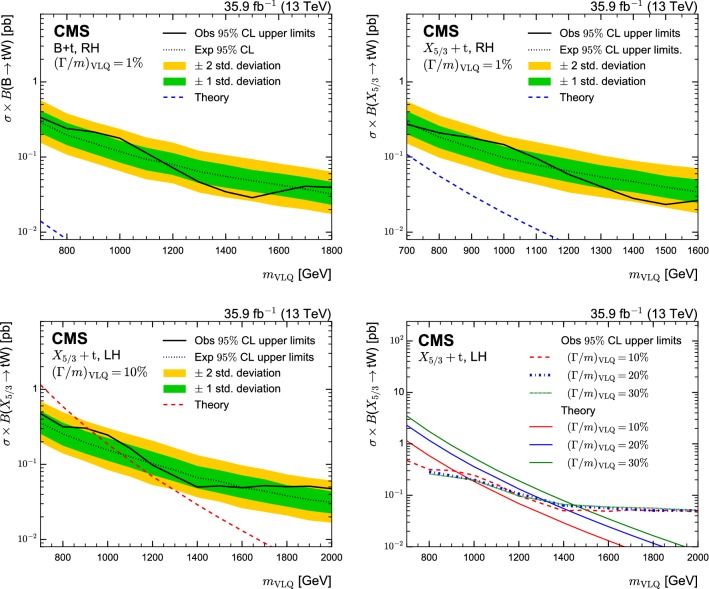
Upper limits at 95% $\text {CL}$ on the product of the VLQ production cross section and branching fraction for the $\mathrm {B}$ +$\mathrm {t}$ and $X_{5/3}$+$\mathrm {t}$ production modes for right-handed VLQ couplings assuming a relative VLQ width of 1% (upper left and right), for the $X_{5/3}$+$\mathrm {t}$ production mode with left-handed VLQ couplings and a 10% relative width (lower left) and a comparison of the observed exclusion limits for left-handed couplings for relative widths of 10, 20, and 30% (lower right). The dashed lines show the theoretical predictions

Figure 7 shows the 95% $\text {CL}$ upper limits on the product of the cross section and branching fraction for the $\mathrm {B}$ +$\mathrm {b}$ production mode for left- and right-handed VLQ couplings and a relative VLQ width of 1% (upper left and upper right), for the left-handed VLQ couplings and a relative VLQ width of 10% (lower left), as well as a comparison of the observed exclusion limits for relative VLQ widths between 10 and 30% (lower right). In Fig. 8, the 95% $\text {CL}$ upper limits on the product of the cross section and branching fraction for the production modes $\mathrm {B}$ +$\mathrm {t}$ (upper left) and $X_{5/3}$+$\mathrm {t}$ (upper right) and right-handed VLQ couplings are shown. The figure also shows the $X_{5/3}$+$\mathrm {t}$ exclusion limits for left-handed VLQ couplings with a 10% relative VLQ width (lower left) and a comparison of the observed exclusion limits for VLQ widths between 10 and 30% for left-handed couplings (lower right). The predicted cross sections for variations of the relative VLQ mass width (dashed lines) are taken from Refs. [55–57]. For a set of VLQ masses the expected and observed 95% $\text {CL}$ upper limits for the $\mathrm {B}$ +$\mathrm {b}$ and the $X_{5/3}$+$\mathrm {t}$ production modes are also given in Table 2 for VLQs with widths of 1% and 10% and left-handed couplings, as well as for widths of 1% and right-handed couplings. The exclusion limits for the $\mathrm {B}$ +$\mathrm {t}$ production mode are similar to those for the $X_{5/3}$+$\mathrm {t}$ production mode.

The obtained exclusion limits range from 0.3 to 0.03$\,\text {pb}$ for VLQ masses between 700 and 2000$\,\text {GeV}$. For VLQs with a relative width of 1% and purely left-handed couplings an increase of about 25% of the 95% $\text {CL}$ upper limits is observed because of the reduced signal acceptance, in comparison to the right-handed couplings. The expected limits for VLQ with relative widths of 10–30% and left-handed couplings only show small differences. Although the predicted cross sections for the SM backgrounds are considerably larger at 13$\,\text {TeV}$, similar exclusion limits on the product of cross section and branching fraction are achieved compared to the results obtained at 8$\,\text {TeV}$ in the more restricted mass range considered in Ref. [32]. However, because of the increase of the VLQ signal cross section at 13$\,\text {TeV}$, with this analysis, the existence of VLQ $\mathrm {B}$ ($X_{5/3}$) quarks with left-handed couplings and a relative width of 10, 20, and 30% can be excluded for masses below 1490, 1590, and 1660$\,\text {GeV}$ (920, 1300, and 1450$\,\text {GeV}$) respectively. The results represent the most stringent exclusion limits for singly produced VLQ in this channel.

**Table 2:** Observed (expected) upper limits at 95% $\text {CL}$ on the product of the cross section and branching fraction for the $\mathrm {B}$ +$\mathrm {b}$ and $X_{5/3}$+$\mathrm {t}$ production modes, for a set of VLQ masses, for VLQs widths of 1% and 10%, and for left-handed and right-handed couplings. The exclusion limits for the $\mathrm {B}$ +$\mathrm {t}$ production mode (not shown) are very similar to those for the $X_{5/3}$+$\mathrm {t}$ mode

$m_\mathrm {{VLQ}}$ (TeV)	$\mathrm {B}$ +$\mathrm {b}$			$X_{5/3}$+$\mathrm {t}$		
	1% LH	10% LH	1% RH	1% LH	10% LH	1% RH
0.8	0.29 (0.36)	0.27 (0.36)	0.25 (0.29)	0.31 (0.27)	0.32 (0.25)	0.21 (0.18)
1	0.29 (0.17)	0.29 (0.19)	0.21 (0.12)	0.25 (0.15)	0.25 (0.16)	0.15 (0.10)
1.2	0.10 (0.10)	0.11 (0.11)	0.07 (0.07)	0.10 (0.09)	0.10 (0.10)	0.06 (0.06)
1.4	0.07 (0.07)	0.06 (0.08)	0.03 (0.05)	0.05 (0.06)	0.05 (0.07)	0.03 (0.05)
1.6	0.05 (0.05)	0.05 (0.06)	0.03 (0.04)	0.04 (0.04)	0.05 (0.05)	0.03 (0.03)
1.8	0.04 (0.04)	0.05 (0.04)	0.03 (0.03)	–	0.05 (0.04)	–

## Summary

A search for singly produced vector-like quarks decaying into a top quark and a $\mathrm {W}$ boson has been performed using the 2016 data set recorded by the CMS experiment at the CERN LHC. The selection is optimised for high vector-like quark masses, with a single muon or electron, significant missing transverse momentum, and two jets with high $p_{\mathrm {T}}$ in the final state. Vector-like quarks in the single production mode can be produced in association with a $\mathrm {t}$ or a $\mathrm {b}$ quark and a forward jet. The latter feature is used to obtain the background prediction in the signal regions from data. The mass of the vector-like quark is reconstructed from the hadronic jets, the missing transverse momentum, and the lepton in the event. Different decay possibilities of the $\mathrm {t}$ and $\mathrm {W}$ are considered. The reach of the search is enhanced by $\mathrm {t}$, $\mathrm {W}$, and $\mathrm {b}$ tagging methods. No significant deviation from the standard model prediction is observed. Upper exclusion limits at 95% confidence level on the product of the production cross section and branching fraction range from around 0.3–0.03$\,\text {pb}$ for vector-like quark masses between 700 and 2000$\,\text {GeV}$. Depending on the vector-like quark type, coupling, and decay width to $\mathrm {t}$ $\mathrm {W}$, mass exclusion limits up to 1660$\,\text {GeV}$ are obtained. These represent the most stringent exclusion limits for the single production of vector-like quarks in this channel.

## Data Availability

This manuscript has associated data in a data repository. [Authors’ comment: Release and preservation of data used by the CMS Collaboration as the basis for publications is guided by the document “CMS data preservation, re-use and open access policy” (https://cms-docdb.cern.ch/cgi-bin/PublicDocDB/RetrieveFile?docid=6032&filename=CMSDataPolicyV1.2.pdf&version=2).]
